# Rab31 promotes metastasis and cisplatin resistance in stomach adenocarcinoma through Twist1-mediated EMT

**DOI:** 10.1038/s41419-023-05596-4

**Published:** 2023-02-13

**Authors:** Ke Chen, Ji Xu, Yu-ling Tong, Jia-Fei Yan, Yu Pan, Wei-jia Wang, Li Zheng, Xiao-xiao Zheng, Can Hu, Xiu Hu, Xian Shen, Wei Chen

**Affiliations:** 1 Department of General Surgery, Sir Run Run Shaw Hospital, School of Medicine, Zhejiang University, Hangzhou, 310016 Zhejiang Province China; 2 Department of General Surgery, The Second Affiliated Hospital and Yuying Children’s Hospital of Wenzhou Medical University, Wenzhou, 325027 Zhejiang Province China; 3 Department of General Practice, The Second Affiliated Hospital of Zhejiang University, School of Medicine, Hangzhou, 310009 Zhejiang Province China; 4 Department of Pharmacy, Affiliated Hangzhou Cancer Hospital, Zhejiang University School of Medicine, Hangzhou, 310002 Zhejiang Province China; 5 Cancer Institute of Integrated Traditional Chinese and Western Medicine, Key Laboratory of Cancer Prevention and Therapy Combining Traditional Chinese and Western Medicine of Zhejiang Province, Zhejiang Academy of Traditional Chinese Medicine, Tongde Hospital of Zhejiang Province, Hangzhou, 310012 Zhejiang Province China; 6 Department of Gastric Surgery, Cancer Hospital of University of Chinese Academy of Sciences, Zhejiang Cancer Hospital, Hangzhou, 310022 Zhejiang Province China; 7 Department of Gastrointestinal Surgery, The Second Affiliated Hospital, Wenzhou Medical University, Wenzhou, 325027 Zhejiang Province China; 8 Institute of Clinical Medicine Research, Zhejiang Provincial People’s Hospital, Hangzhou Medical College, Hangzhou, 310014 Zhejiang Province China

**Keywords:** Tumour biomarkers, Cancer therapeutic resistance

## Abstract

Stomach adenocarcinoma (STAD) is one of the leading causes of cancer-related death globally. Metastasis and drug resistance are two major causes of failures in current chemotherapy. Here, we found that the expression of Ras-related protein 31 (Rab31) is upregulated in human STAD tissues and high expression of Rab31 is closely associated with poor survival time. Furthermore, we revealed that Rab31 promotes cisplatin resistance and metastasis in human STAD cells. Reduced Rab31 expression induces tumor cell apoptosis and increases cisplatin sensitivity in STAD cells; Rab31 overexpression yielded the opposite result. Rab31 silencing prevented STAD cell migration, whereas the overexpression of Rab31 increased the metastatic potential. Further work showed that Rab31 mediates cisplatin resistance and metastasis via epithelial-mesenchymal transition (EMT) pathway. In addition, we found that both Rab31 overexpression and cisplatin treatment results in increased Twist1 expression. Depletion of Twist1 enhances sensitivity to cisplatin in STAD cells, which cannot be fully reversed by Rab31 overexpression. Rab31 could activate Twist1 by activating Stat3 and inhibiting Mucin 1 (MUC-1). The present study also demonstrates that Rab31 knockdown inhibited tumor growth in mice STAD models. These findings indicate that Rab31 is a novel and promising biomarker and potential therapeutic target for diagnosis, treatment and prognosis prediction in STAD patients. Our data not only identifies a novel Rab31/Stat3/MUC-1/Twist1/EMT pathway in STAD metastasis and drug resistance, but it also provides direction for the exploration of novel strategies to predict and treat STAD in the future.

## Introduction

As the fifth most common malignant tumor, stomach cancer accounts for 5.6% of all new cancer cases and has the fourth highest cancer mortality rate worldwide [1]. This malignancy encompasses several histological types, of which stomach adenocarcinoma (STAD) is the most common one, responsible for ~95% of all cases [2]. Significant progress has been made in the treatment of STAD in the past decades and cisplatin is the primary chemotherapeutic agent for STAD patients [3]. However, the development of drug resistance reduces the effectiveness of cisplatin, resulting in local infiltration and distant metastases. Consequently, the prognosis of STAD remains dismal with the 5-year survival rate less than 30% [4]. Accordingly, understanding the molecular mechanisms underlying the STAD metastasis and chemoresistance is urgently required for identifying novel drug targets and developing more effective therapeutic strategies.

Ras-related protein 31 (Rab31, also termed Rab22B), a member of the Rab family derived from monomeric GTP-binding proteins, plays a significant role in regulating intracellular vesicle trafficking between the Golgi/TGN and endosomes [5–7]. Accumulating evidence reveals that dysregulation of Rab31 is involved in tumor development and progression. For example, Rab31 expression in hepatocellular carcinoma tissues is remarkably higher than that in adjacent liver tissues, and Rab31 is a novel prognosis biomarker in patients with hepatocellular carcinoma [8]. Furthermore, Rab31 promotes cell proliferation and migration, inhibiting cell apoptosis in glioblastoma and cervical cancer cell lines. Rab31 silencing suppresses tumor growth in vivo [9]. Notably, Rab31 regulates the switch between an invasive and proliferative phenotype in breast cancer cells, which depends on its expression level. Increased expression of Rab31 is related to enhanced proliferation, leading to a decreased invasive capacity of breast cancer cells [10]. Recently, Rab31 was reported to function as an oncogene in gastric cancer tumorigenesis and may serve as a therapeutic target in gastric cancer, which is in line with our previous study [7, 11]. However, the underlying molecular mechanism of Rab31 in STAD progression remains to be fully understood.

Epithelial-mesenchymal transition (EMT) plays an important role in promoting metastasis and drug resistance in various cancers including STAD [12]. During EMT, cancer cells lose epithelial characteristics and gain a mesenchymal, highly migratory and invasive phenotype [13]. Twist-related protein 1 (Twist1), a basic helix-loop-helix transcription factor associated with EMT, is related to metastasis of many cancer cell types [14]. Moreover, Twist1 was reported to facilitate invasion and EMT in gastric adenocarcinoma [15]. Up-regulation of Twist1 was associated with resistance to both conventional chemotherapy and target agents in breast, prostate, hepatocellular carcinoma, and lung cancer cell lines [16]. MUC-1 is a heterodimeric protein that has abnormal high expression in various human cancers. Its C-terminal transmembrane subunit (MUC1-C) is oncogenic by associating with receptor tyrosine kinases, activating the downstream signaling effectors [17]. Furthermore, MUC1-C binds to Twist1 and forms an autoregulatory loop with Twist1, regulating EMT and acquired paclitaxel resistance in triple-negative breast cancer [18].

In the present study, we demonstrate that Rab31 is overexpressed in STAD and is significantly related to poor overall survival (OS). In addition, Rab31 was found to promote cell migration, contribute to Twist1-mediated EMT, and induce cisplatin resistance in STAD. Depletion of Rab31 suppressed STAD cell migration and EMT and enhanced sensitivity to cisplatin in vitro. Furthermore, Rab31-overexpressing cells conferred the opposite effects. Our results revealed that Rab31 promotes tumor metastasis and cisplatin resistance in STAD through Twist1-mediated EMT, which suggests that Rab31/Stat3/MUC-1/Twist1 pathway is a promising therapeutic target to overcome resistance to cisplatin and metastasis in STAD.

## Results

### High Rab31 expression is associated with poor prognosis in STAD patients

**Fig. 1:**
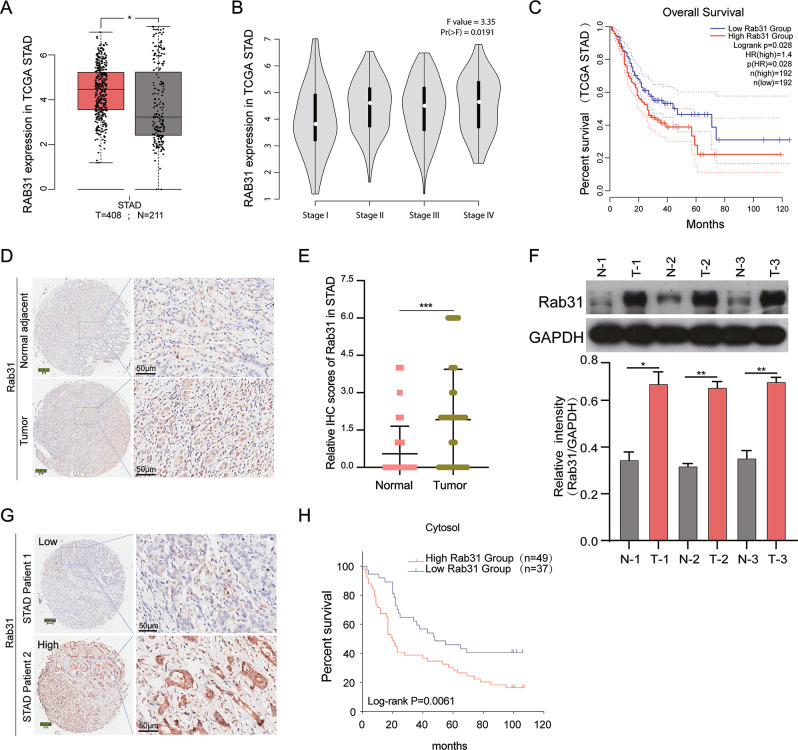
High expression levels of Rab31 were observed in the STAD clinical samples and predicted poor prognosis. **A** The expression of Rab31 was significantly higher in 408 STAD tissues than in 211 normal tissues. STAD: Stomach adenocarcinoma; T: Tumor; N: Normal. *P < 0.05. **B** The expression of Rab31 was associated with tumor stage in STAD. **C** The high expression of Rab31 was associated with poor overall survival. **D, E** Immunohistochemical staining showed that the expression levels of Rab31 were significantly upregulated in clinical STAD tissue samples compared with normal tissues. ***P < 0.001. Scale bar: 50 μm. **F** Rab31 was overexpressed in STAD compared with expression in 3 pairs of corresponding adjacent normal tissues using western blot. *P < 0.05 and **P < 0.01. **G**, **H** a high level of Rab31was associated with poor survival and may be a biomarker in STAD patients. Scale bar: 50 μm.

We evaluated the mRNA levels of Rab31 by analyzing the RNA-Seq data of 408 STAD tissues and 211 normal tissues retrieved from The Cancer Genome Atlas (TCGA) database. Rab31 expression was significantly elevated in STAD tumor tissues compared with normal tissues (Fig. 1A). In addition, the expression of Rab31 increased with STAD progression from stage I to stage IV, with the highest expression level of Rab31 in stage IV (Fig. 1B). Furthermore, STAD patients with high Rab31 level is associated with poor OS (Fig. 1C).

To further assess the expression of Rab31 in STAD, we examined its protein levels in human STAD tissue microarray (HStmA180Su15) (Shanghai Outdo Biotech Co., Ltd) by immunohistochemistry. Rab31 was significantly upregulated in tumor tissues compared to adjacent normal tissues (Fig. 1D, E). We also examined the Rab31 protein levels from clinically collected tissues by western blot and confirmed that Rab31 was significantly increased in STAD tissues compared to that in the corresponding normal tissues (Fig. 1F). In addition, we compared the average survival rate of 49 Chinese STAD patients with high level of Rab31 to the group of 37 Chinese patients having low Rab31. The 10-year survival rate of patients with high Rab31 was 43%, while the 10-year survival rate of low Rab31 group was 57%, further confirming that high Rab31 is closely correlated with poor survival in STAD patients (Fig. 1G-H). These data indicate that Rab31 expression is upregulated in STAD tissues and its high expression correlates with poor prognosis in STAD patients, which lead us to hypothesize that Rab31 might play an important role in the progression of STAD.

### Rab31 promotes cisplatin resistance in human STAD cells

**Fig. 2:**
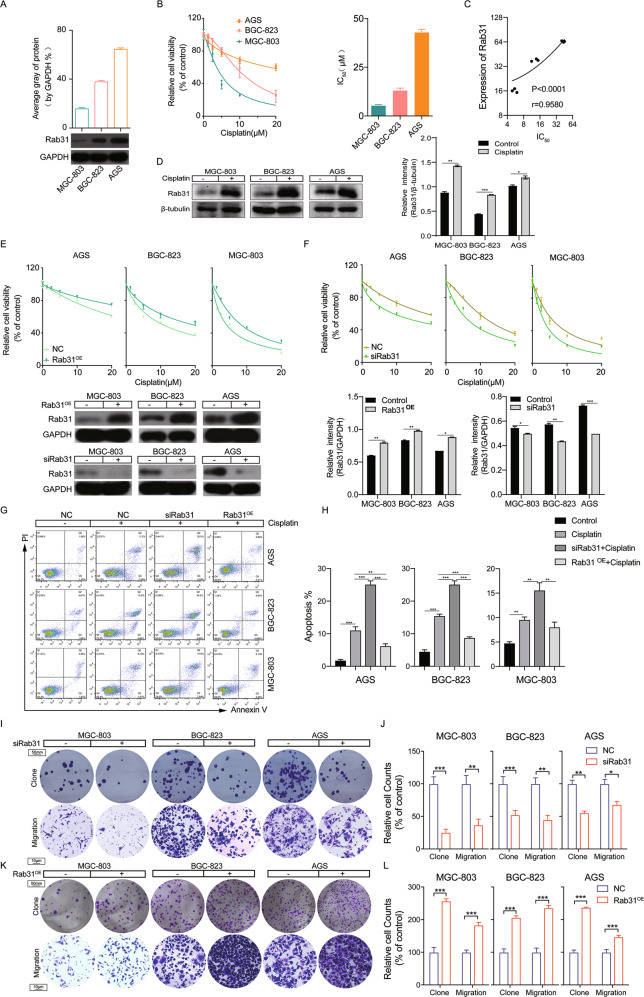
Rab31 promotes cell migration and regulated cisplatin sensitivity in human STAD cells. **A** The expression levels of Rab31 in three STAD cell lines. **B** Cell proliferation was determined by the CCK-8 assay. IC_50_ were calculated. **C** The relationship between Rab31expression and IC_50_ in STAD cells. **D** Cells were incubated with cisplatin and the expression of Rab31 was determined by western blot. *P < 0.05, **P < 0.01 and ***P < 0.001, versus control. **E**, **F** CCK-8 assays were performed to determine the resistance or sensitivity to cisplatin. Transfection efficiency was assessed by western blot. *P < 0.05, **P < 0.01 and ***P < 0.001, versus control. **G** Quantification of apoptosis by flow cytometric analysis of Annexin-V and PI staining after treatment with cisplatin. A representative flow profile is presented. **H** Summary of the percentage for Annexin V-positive cells. **P < 0.01, ***p < 0.001. **I** The colony formation assay (Scale bar: 50 μm) and transwell migration analysis (Scale bar: 10 μm.) showed that Rab31 knockdown markedly inhibited proliferation and migration. **K** The colony formation assay (Scale bar: 50 μm) and transwell migration analysis (Scale bar: 10 μm) showed that Rab31 overexpression markedly promoted proliferation and migration. **J**, **L** Quantitative analysis of colony formation and transwell migration assay in groups. *P < 0.05, **P < 0.01, ***P < 0.001.

Given that resistance to cisplatin is one main reason of poor STAD prognosis, we sought to determine whether Rab31 is involved in cisplatin resistance in STAD. We utilized three human STAD cell lines: MGC-803, BGC-823, and AGS. The expression of Rab31 was the lowest in MGC-803 cells and the highest in AGS cells (Fig. 2A). The cell viability of all three human STAD cells decreased with cisplatin treatment in a dose-dependent manner (Fig. 2B, left). Interestingly, AGS cells showed the highest resistance to cisplatin with half-maximal inhibitory concentrations (IC_50_) of 43.08 μM, while MGC-803 cells had lowest IC_50_ 5.46 μM (Fig. 2B, right). There is a significant positive correlation between IC_50_ and Rab31 expression levels (*r*^2^ = 0.9580, *P* < 0.0001; Fig. 2C). These results suggested that Rab31 expression might restrict the anti-tumor activity of cisplatin in STAD cells.

To test whether Rab31 inhibits cisplatin effectiveness, we first examined how Rab31 expression is changed in cells treated with cisplatin. The western blot results demonstrated that the Rab31 protein levels were significantly increased in all three human STAD cell lines when treated with cisplatin (Fig. 2D). We then increased Rab31 levels by transfection of cells with Rab31 expression plasmid (Rab31^OE^) and reduced Rab31 by siRNA. Cells overexpressing Rab31 (Rab31^OE^) showed a significant increase in Rab31 protein levels and Rab31 siRNA significantly reduced Rab31 expression in all three STAD cells (Fig. 2E, F, bottom). Rab31 overexpression caused upward shift of the dose-response curve in all three STAD cell lines treated with cisplatin, suggesting that increase in Rab31 enhance cell viabilities of cisplatin-treated STAD cells (Fig. 2E, top). Additionally, reduction in Rab31 expression in all three STAD cell lines resulted in downward shift of the dose-response curve of cisplatin treatment, indicating that low Rab31 expression can increase cisplatin sensitivity (Fig. 2F, top). The effects of Rab31 expression on cisplatin resistance was further examined by measuring the cell apoptosis with Annexin V/PI staining. The early and late apoptosis was induced in all STAD cells treated with cisplatin at IC50 concentration for 48 h (Fig. 2G). Rab31 knockdown significantly increased cisplatin-induced apoptosis, with more cells in the late apoptosis (Annexin+/PI+) (Fig. 2G). Interestingly, such effects were most evident in AGS cells that have high endogenous Rab31 levels (Fig. 2H). Furthermore, overexpression of Rab31 reduced cisplatin-induced apoptosis in all these three cell lines (Fig. 2H).

### Rab31 promotes STAD tumor metastasis

Next, we studied how Rab31 affects the long-term proliferative capacity and metastasis in STAD cells by colony formation assay and transwell assay. Rab31 knockdown inhibited the colony formation in all three STAD cell lines tested, while Rab31 overexpression enhanced the formation of tumor colonies (Fig. 2I, K, top). In the transwell assay, Rab31 silencing prevented STAD cell migration, whereas the overexpression of Rab31 increased the metastatic potential (Fig. 2I, K, bottom). The results suggest that Rab31 may promote clonogenic growth and metastasis of human STAD cells.

### Rab31 promotes metastasis and cisplatin resistance through EMT

**Fig. 3:**
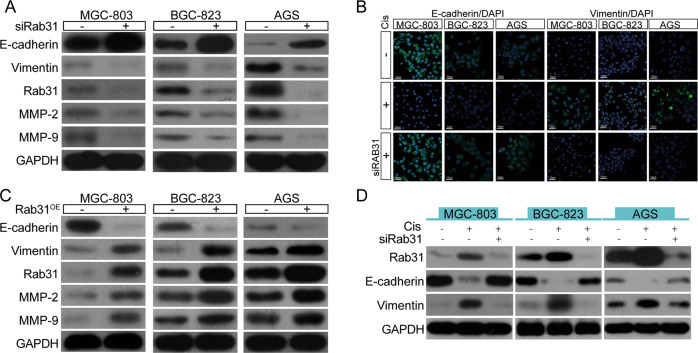
Rab31 promotes metastasis and depresses cisplatin sensitivity via EMT. **A** STAD cells were transfected with control siRNA and Rab31 siRNA for 48 h, and the expression of indicated proteins were detected by western blot. **B** Expression of E-cadherin and vimentin in control group, cisplatin group and Rab31 siRNA plus cisplatin group was analyzed by immunofluorescence. Scale bar: 10 μm. **C** STAD cells were transfected with empty vector and Rab31 for 48 h, and the expression of indicated proteins were detected by western blot. **D** EMT markers in control group, cisplatin group and Rab31 siRNA plus cisplatin group were detected by western blot.

In human cancers, EMT is a key cell process that is associated with tumor progression, metastasis, and resistance to therapy, during which epithelial cells acquire mesenchymal characteristics [19]. Recently, Rab31 was found to be involved in EMT in oral squamous cell carcinoma [20]. To determine whether EMT was involved in Rab31-induced metastasis and resistance to cisplatin in STAD, we measured the expression of epithelial cell markers and mesenchymal cell markers when Rab31 expression was altered in human STAD cell lines. As shown in Fig. 3A, Rab31 knockdown in all three STAD cell lines caused an increase in the protein expression levels of epithelial marker E-cadherin and a reduction in the expression of the mesenchymal markers (vimentin, MMP-2 and MMP-9). In contrast, Rab31 overexpression resulted in EMT characterized with increased expression of vimentin, MMP-2, and MMP-9, and loss of E-cadherin expression (Fig. 3C).

We then explored how cisplatin treatments affect EMT. When human STAD cells were treated with cisplatin at IC50 concentration for 24 hours, expression of E-cadherin was reduced and expression of and vimentin was increased, suggesting that cisplatin induces EMT (Fig. 3B, D). Rab31 siRNA reversed the cisplatin-induced EMT process as evidenced by the recovered expression of E-cadherin and decreased expression of vimentin by immunofluorescence (Fig. 3B) and Western Blot (Fig. 3D). The results suggest that increased Rab31 expression during cisplatin treatment may promote EMT, which enhances cell migration and metastatic ability of STAD cells, leading to cisplatin resistance.

### Rab31 promotes Twist1-mediated EMT by targeting MUC-1

**Fig. 4:**
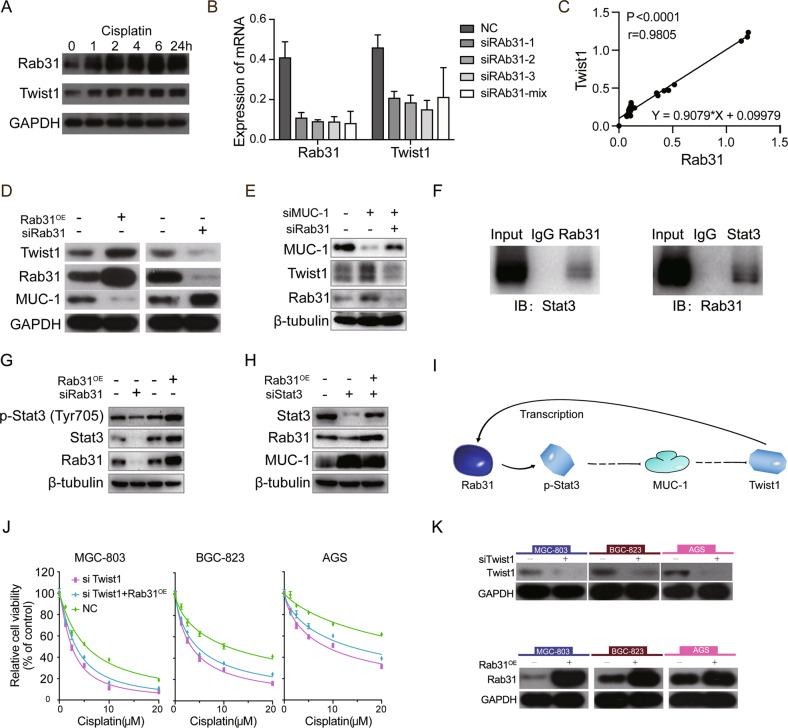
Rab31 promotes Twist1-mediated EMT by targeting MUC-1. **A** western blot showed that Rab31 and Twist1 were upregulated after cisplatin treatment in AGS cells. **B** The mRNA expression of Rab31 and Twist1 were detected by qRT-PCR. **C** The relationship between Twist1 expression and Rab31 expression. **D** Western blot analysis of Twist1, Rab31 and MUC-1 for the indicated groups in AGS cells. **E** Western blot analysis of MUC-1, Twist1 and Rab31 for the indicated groups in AGS cells. **F** The interaction between Rab31 and Stat3 were tested using coimmunoprecipitation. **G** Western blot analysis of p-Stat3, Stat3 and Rab31 for the indicated groups in AGS cells. **H** Western blot analysis of Stat3, Rab31 and MUC-1 for the indicated groups in AGS cells. **I** The mechanism of Rab31 regulate Twist1. **J** CCK-8 assays were performed to determine the sensitivity to cisplatin in the indicated groups. **K** Transfection efficiency was assessed by western blot.

The EMT process is coordinated by a set of EMT transcription factors (EMT-TFs). Recently, EMT-TFs have been suggested to mediate chemoresistance. For example, EMT-TFs such as TWIST1 and ZEB1 have been reported to drive EMT-mediated resistance to EGFR inhibitors in EGFR-mutant non-small cell lung cancer [21, 22]. To identify the potential EMT-TFs involved in the Rab31-medaited cisplatin resistance in STAD, the correlation between the expression of EMT-TFs and Rab31 were calculated using GEPIA online tool. The results showed that EMT-TFs (TWIST1, SNAI1, SNAI2, ZEB1, and ZEB2) had a positive correlation with Rab31 (Fig. S1). Numerous studies have reported that Twist1 was overexpressed in STAD compared with noncancerous tissues and high expression of Twist1 is correlated with poor progression [15, 23, 24]. We then tested whether Twist1 is the key player that can connect Rab31 with EMT and cisplatin resistance. Western Blot results showed that cisplatin treatment in AGS cells increased the expression of Rab31 and Twist1 in a time-dependent manner (Fig. 4A). Moreover, Rab31 knockdown decreased the mRNA expression of Twist1 in AGS cells (Fig. 4B). The expression levels of Twist1 and Rab31 are positively correlated (*r*^2^ = 0.9079, *P* < 0.0001; Fig. 4C).

Silencing MUC1-C could inhibit Twist1 and thereby reverses the paclitaxel resistance in triple-negative breast cancer [18]. Here we examined whether MUC-1 is involved in the Rab31-Twist1 signaling axis in STAD cells. Overexpression of Rab31 resulted in upregulation of Twist1 and downregulation of MUC-1 protein levels, while Rab31 knockdown reduced Twist1 and increased MUC-1 in AGS cells (Fig. 4D). Furthermore, the correlation between MUC-1 and Twist1/Rab31 was further confirmed using starBase (http://starbase.sysu.edu.cn/). As shown in Fig. S2C, D, the expression levels of MUC-1 were negatively correlated with that of Twist1/Rab31. Moreover, MUC-1 knockdown increased the expression of Twist1 and Rab31 in AGS cells, while MUC-1/Rab31double-knockdown reversed these changes, as assessed through western blot (Fig. 4E). These data indicated that Rab31 might activate Twist1 by suppressing MUC-1 (Fig. 4I). However, coimmunoprecipitation showed that Rab31 did not directly interact with Twist1 or MUC-1 in AGS cells (Fig. S2A). Previous study reported that MUC-1 can operate through Stat3. Our data showed that Rab31 directly interact with Stat3 (Fig. 4F). Furthermore, Rab31 knockdown resulted in downregulation of Stat3 and p-Stat3 protein levels, while overexpression of Rab31 increased the expression of Stat3 and p-Stat3 in AGS cells (Fig. 4G). Moreover, Stat3 knockdown downregulation of Stat3 level and increased the expression of MUC-1 in AGS cells, while overexpression of Rab31 reversed these changes, as assessed through western blot (Fig. 4H). Our results indicated that MUC-1 is a downstream target of Stat3.

Next, we investigated how Rab31/Stat3/MUC-1/Twist1 signaling is involved in cisplatin resistance in STAD. Cisplatin treatment upregulated the expression of Twist1 and vimentin, and downregulated E-cadherin expression, Twist1 knockdown reversed these changes, as assessed through western blot (Fig. S3B). Notably, CCK-8 and EDU assay revealed that twist1 knockdown enhanced cisplatin-induced antiproliferation effects on STAD cell lines (Fig. S3C–E). We found that Twist1 silencing enhanced cisplatin-induced death in STAD cell lines (Fig. 4J, K). These results indicated that Twist1-mediated EMT is associated with STAD cells metastasis and cisplatin resistance. Importantly, Rab31 overexpression only slightly reversed the effect of Twist1 knockdown (Fig. 4J, K), indicating that Twist1 is the signaling molecule downstream of Rab31 to confer cisplatin resistance. All these results support a Rab31-Twist1 pathway in driving EMT and cisplatin resistance in STAD. Moreover, we also analyzed the effect of Rab31 on the crucial factors regulating Twist1 expression. The results showed that Rab31 knockdown increased the expression of miR15 and miR373, overexpression of Rab31 decreased the expression of miR15 and miR373 (Fig. S6). MiR15 and miR373 may play an important role in Rab31–Twist1 pathway.

### Rab31 silencing inhibits tumor growth in vivo

**Fig. 5:**
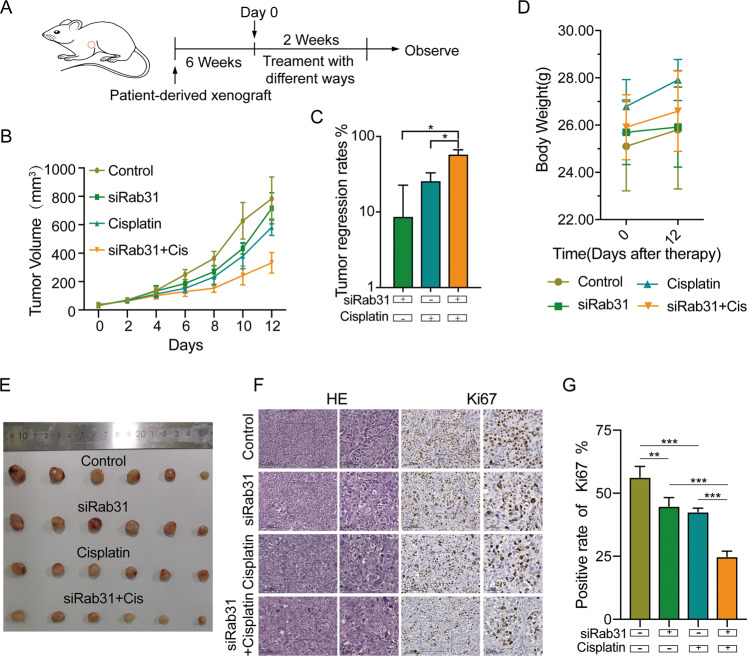
Rab31 depresses sensitivity to cisplatin in a PDX model of human STAD. **A** Flowchart of our animal experiments. **B** Volume of tumor xenografts was measured every other day in the different treatment groups. **C** Tumor regression rates (*p < 0.05, cisplatin plus Rab31 siRNA vs. Rab31 siRNA alone; *P < 0.05, cisplatin plus Rab31 siRNA vs. cisplatin alone). **D** The body weight was measured in the different treatment groups. **E** Representative images of tumors at day 12. **F** Rab31 levels were significantly associated with expression of Ki67. Representative images of tumors at day 12 are shown. Scale bar: 125 μm (left panel); 50 μm (right panel). **G** Positive rate of Ki67 (**P < 0.01, control vs. Rab31 siRNA alone; ***P < 0.001, control vs. cisplatin alone; ***P < 0.001, cisplatin plus Rab31 siRNA vs. Rab31 siRNA alone; ***P < 0.001, cisplatin plus Rab31 siRNA vs. cisplatin alone).

Lastly, we extended to examine the influence of Rab31 expression levels on tumor growth in vivo by employing a patient-derived xenograft (PDX) model in nude mice using human STAD tissue. Animal experiments were performed according to flowchart as shown in Fig. 5A. Rab31 knockdown yields the equivalent effects as cisplatin treatment for inhibiting the tumor growth compared to the control (Fig. 5B, E). Treatment with cisplatin along with Rab31 siRNA conferred the highest inhibition on tumor growth (Fig. 5B, C). There was no difference in body weight among the different treatment groups, indicating that the changes in tumor size are not due to changes in body weight. (Fig. 5D). In addition, the tumor cell proliferation was measured by HE and Ki67 staining of tumor tissue. Simultaneous treatment with cisplatin and Rab31 knockdown significantly reduced tumor cell proliferation (Fig. 5F, G), suggesting that Rab31 is a potential therapeutic target that can be paired with cisplatin treatment for STAD.

## Discussion

Stomach cancer remains a globally important disease, responsible for over 1 million estimated new cases and 769,000 deaths in 2020 [1]. Although the incidence and mortality rates of stomach cancer have declined over the past century, the total stomach cancer cases will grow due to the increase in ageing populations [25]. The burden of stomach cancer remains high, accounting for 20% of the total disability-adjusted life-years worldwide [26]. Despite advances in the target treatment in the past few decades, surgery is considered to be the only curative therapy until now. Furthermore, the development of metastasis and resistance to chemotherapy are the main obstacles that restrict the clinical efficacy of cancer treatment [27]. Therefore, new strategies that are not prone to drug resistance development are urgently required.

Recent studies have shown that Rab31 is an oncogene for numerous cancers, and Rab31 overexpression is related to cancer progression and poor prognosis [20, 28, 29]. However, the role of Rab31 in STAD progression is rarely studied and poorly understood. In our study, the expression of Rab31 was upregulated in STAD samples compared with the adjacent normal tissues (Fig. 1D–F), which is in line with previous study [11]. In addition, the expression of Rab31 elevated along with STAD progression from stage I to stage IV (Fig. 1B). Meanwhile, STAD patients with high-Rab31 expression was closely related to poor OS (Fig. 1C, H, I). All these evidences indicates that Rab31 is a potential biomarker for the diagnosis and prognosis of STAD.

Resistance to therapy is a challenge for all cancer treatments, and the mechanism of drug resistance is still not fully understood [30]. Rab31 was reported to be downregulated in docetaxel-, tamoxifen-, and doxorubicin-resistant MCF-7 cell lines. Diminished expression of Rab31 elevates resistance toward tamoxifen, while ectopic expression of Rab31 significantly increases tamoxifen sensitivity [31]. These results suggest that Rab31 downregulation contributes to the development of drug resistance in breast cancers. In this paper, we investigated the role of Rab31 in cisplatin resistance in STAD. Different from the results obtained in breast cancer cells, we found that Rab31 expression is enhanced with cisplatin treatment and Rab31 increased cisplatin resistance in STAD cells (Fig. 2D–H). The in vivo work using PDX model provided further convincing evidence that Rab31 is an effective therapeutic target to treat STAD. We showed that Rab31 knockdown along with cisplatin significantly inhibited tumor growth compared with the group treated with cisplatin alone (Fig. 5B, E).

We then identified the downstream cell processes and signaling molecules that can be regulated by Rab31 in STAD. We demonstrated that silencing Rab31 inhibited cell migration and overexpression of Rab31 promoted cell migration (Fig. 2I, K, bottom). In addition, we found that Rab31 overexpression markedly enhance EMT evidenced by the downregulation of E-cadherin and increased expression of vimentin, MMP-2, and MMP-9, whereas Rab31 knockdown suppress EMT (Fig. 3A, C). Our results demonstrated, for the first time, that Rab31 can promote the metastasis and EMT in STAD cells.

The EMT has been reported to be a link between cancer metastasis and drug resistance, because tumors with drug resistance are apt to metastasize [32]. Our results revealed that Rab31 stimulates cisplatin resistance by regulating EMT in STAD cells (Fig. 3B, D). The EMT transcription factor Twist1 is overexpressed in gastric cancer and is associated with an increased migration and decreased sensitivity to cell death [16, 33]. We found that cisplatin upregulated the expression of Twist1, and Twist1 knockdown enhanced cisplatin-induced death in STAD cell lines (Fig. 4A, G). Moreover, decrease in Rab31 expression suppressed the mRNA expression of Twist1 (Fig. 4B). And the expression of Twist1 was positively correlated with Rab31 (Fig. 4C). These results indicate that Twist1 expression is downstream of Rab31. Given that coimmunoprecipitation showed no direct interaction between Twist1 and Rab31, other proteins exist to bridge the connection between Rab31 and Twist1.

Previous finding has reported that MUC1-C forms a complex with ERα on the ERα-responsive Rab31 promoter and activates Rab31 gene transcription in an estrogen-dependent manner [34]. Moreover, Hata et al. [18] reported that MUC1-C binds to Twist1, forms an autoregulatory loop with Twist1 and regulates EMT, and targeting MUC1-C could inhibit Twist1, reverses the paclitaxel resistance in triple-negative breast cancer. In current study, we found that Rab31 activated Twist1 by suppressing MUC-1. Rab31 functions as a master regulator of EMT and resistance to cisplatin in STAD. Furthermore, Rab31 inhibited MUC-1 via activating Stat3.

In conclusion, this study revealed the aberrant high expression of Rab31 was in the STAD tumors, the expression of Rab31 increased along with STAD progression, and high Rab31 expression was related to the poor OS of STAD patients. Meanwhile, our data indicated Rab31 as a novel pro-metastatic factor and a novel predictive biomarker for cisplatin resistance in STAD patients. Rab31 promotes metastasis and resistance to cisplatin therapy of the STAD cells via Stat3/MUC-1/Twist1-mediated EMT. Our data not only identifies a novel pathway in STAD metastasis and drug resistance, but it also directs us to explore novel strategies to predict and treat STAD in the future.

## Materials and methods

### Drugs and reagents

Cisplatin (cat. no. S1166) was purchased from Selleck Chemicals. The antibodies used for western blot, immunohistochemistry, and immunofluorescence staining were as following: anti-Rab31 antibody (cat. no. H00011031-M03) was purchased from Abnova; anti-MMP-2 antibody (cat. no. 48587) was purchased from SAB; anti-Ki67 antibody (cat. no. ab16667) and anti-MUC-1 antibody (cat. no. ab109185) were purchased from Abcam; anti-β-tubulin antibody (cat. no. ET1602-4) was purchased from HUABIO; anti-vimentin antibody (5G3F10, cat. no. 3390), anti-E-cadherin antibody (24E10, cat. no. 3195), anti-MMP-9 antibody (D6O3H, cat. no. 13667), anti-Twist1 antibody (cat. no. 46702), anti-GAPDH antibody (14C10, cat. no. 2118), anti-Stat3 antibody (124H6, cat. no. 9139), anti-Phospho-Stat3 (Tyr705) antibody (D3A7, cat. no. 9145), and HRP-linked secondary antibody anti-rabbit lgG (cat. no. 7074) and anti-mouse lgG (cat. no. 7076) were obtained from Cell Signaling Technology.

### Patients and sample collection

Fresh STAD tissue samples and paired adjacent tissue samples were obtained from three different STAD patients undergoing surgical procedures at Zhejiang Cancer Hospital. All samples were stored at −80 °C until required. Before the use of these clinical materials for research, written consents from all patients and approval of Zhejiang Cancer Hospital Ethic Review Committees were obtained.

### Cell culture

Human STAD cell lines (MGC-803, BGC-823 and AGS) were purchased from the Shanghai Institute of Biochemistry and Cell Biology (Shanghai, China). MGC-803, BGC-823 and AGS cells were maintained in RPMI-1640 medium supplemented with 10% fetal bovine serum (FBS). All cell lines were cultured in a humidified atmosphere at 37 °C with 5% CO_2_. The source of cell lines was recently authenticated by STR profiling and tested for mycoplasma contamination.

### Western blot

Western blot analysis was operated as previously described [35]. Briefly, the protein samples were separated by SDS-PAGE and transferred onto PVDF membranes. Then, the membranes were blocked and incubated with primary antibodies followed by secondary antibodies. Finally, protein bands were incubated with ECL reagent and visualized on autoradiography film.

### Data collection and analysis

The data were collected from GEPIA (Gene Expression Profiling Interactive Analysis; http://gepia.cancer-pku.cn/) and UALCAN (http://ualcan.path.uab.edu/index.html), TCGA (The Cancer Genome Atlas) online analysis tool [36, 37]. The Rab31 expression profiles for sample types (tumor and normal) and stage were obtained. Moreover, the association in Rab31 expression level and prognosis was also obtained.

### Transfection

Cells (1 × 10^5^ per well) were seeded in six-well plates. The following day, cells were transfected with indicated small interfering RNA (siRNA) or plasmids using Lipofectamine 2000 (Invitrogen, Carlsbad, CA), according to the manufacturer’s instructions. The specific siRNA against Rab31 (cat. no. sc-76327), Twist1 (cat. no. sc-38604), and scramble siRNA (sc-37007) were purchased from Santa Cruz Biotechnology. The Rab31 plasmid was constructed and validated by GenePharma (Shanghai, China) according to the NM sequence (NM_006868). The sequences of the Rab31 siRNAs and Twist1 siRNAs are as follows:

Rab31-homo-76327A, 5′ CAGCUGUUAUCGUGUAUGATT 3′

5′ UCAUACACGAUAACAGCUGTT 3′

Rab31-homo-76327B, 5′ GAACUGAUUCCUACUGAAATT 3′

5′ UUUCAGUAGGAAUCAGUUCTT 3′

Rab31-homo-76327C, 5′ CAAGCCAGUCAGAGGAUAATT 3′

5′ UUAUCCUCUGACUGGCUUGTT 3′

Twist1-homo-38604A, 5′ CUCUGGAGCUGGAUAACUATT 3′

5′ UAGUUAUCCAGCUCCAGAGTT 3′

Twist1-homo-38604B, 5′ GCAUCACUAUGGACUUUCUTT 3′

5′ AGAAAGUCCAUAGUGAUGCTT 3′

Twist1-homo-38604C, 5′ CAGAGGAACUAUAAGAACATT 3′

5′ UGUUCUUAUAGUUCCUCUGTT 3′

MUC-1-homo-389, 5′ GCCUCUCCAAUAUUAAGUUTT 3′

5′ AACUUAAUAUUGGAGAGGCTT 3′

MUC-1-homo-447, 5′ CCGAGAAGGUACCAUCAAUTT 3′

5′ AUUGAUGGUACCUUCUCGGTT 3′

MUC-1-homo-725, 5′ GGGAUACCUACCAUCCUAUTT 3′

5′ AUAGGAUGGUAGGUAUCCCTT 3′

Stat3-homo-1729, 5′ GGGACCUGGUGUGAAUUAUTT 3′

5′ AUAAUUCACACCAGGUCCCTT 3′

Stat3-homo-1272, 5′ CCCGGAAAUUUAACAUUCUTT 3′

5′ AGAAUGUUAAAUUUCCGGGTT 3′

Stat3-homo-1878, 5′ GGUACAUCAUGGGCUUUAUTT 3′

5′ AUAAAGCCCAUGAUGUACCTT 3′

### Flow cytometry analysis

Apoptosis was examined using Flow cytometry (BD Biosciences, Franklin Lakes, NJ, USA). Fluorescein isothiocyanate (FITC)-conjugated annexin-V and a Propidium Iodide (PI) kit (BD Biosciences, San Jose, CA, USA) were used according to the manufacturer’s instructions. The data were analyzed with FlowJo (Ashland, OR, USA). The details are described in our previous study [38].

### Colony formation assay

After cell transfection, cells (2000 per well) were seeded in 6‐well plates and cultured for 14 days to allow colony formation. Next, the cells were stained with 1% crystal violet (AMRESCO), washed with tap water, and then photographed for counting.

### Transwell migration assay

Cells (5 × 10^4^ per well) were seeded in the upper chambers (24-well insert, 8 mm, Corning, NY) of transwell inserts. They were allowed to migrate through the pores in the transwell membrane during incubation at 37 °C for 24 h. Thereafter, cells growing on the bottom side of the membrane were fixed in 4% paraformaldehyde, stained with 0.1% crystal violet, and counted.

### Cell viability assay

Cell viability was measured by Cell Counting Kit-8 assay (CCK8; Dojindo; Kumamoto, Japan) according to the manufacturer’s instruction. After cell transfection, cells (5 × 10^3^ per well) were seeded in 96-well plates and treated with cisplatin for 48 h. And optical density determination was measured by A MAX II microplate reader (Dynex Technologies, Chantilly, VA) at 450 nm.

### Immunohistochemistry (IHC) and immunofluorescence staining

IHC study was performed as previously described [39]. STAD tissues were embedded in paraffin. Then, the sections were deparaffinized and rehydrated, then incubated with primary antibodies followed by secondary antibodies. Protein expression was visualized using 3,3′-diaminobenzidine and counterstained with hematoxylin. In order to avoid visual bias, three different pathologists evaluated Rad31 IHC score independently.

Immunofluorescence was also performed as previously described [35]. Briefly, STAD cells were fixed with 4% paraformaldehyde and blocked with 5% BSA. Next, cells were incubated with primary antibodies followed by secondary antibodies. Finally, cells were incubated with 0.1% DAPI and observed under an inverted fluorescence microscope (Olympus, Tokyo, Japan).

### qRT-PCR

Total RNA from STAD cell samples were isolated using TRIzol reagent (Invitrogen Life Technologies), according to the manufacturer’s instructions. Then, the RNA was reverse-transcribed into cDNA using the TaqMan Reverse Transcription Kit (Applied Biosystems) as previously described [35]. GAPDH was used as the housekeeping gene. The primers used in qRT-PCR are as follows:

Rab31,

Rab31-F 5′ CTCGAATTCAATGATGGCGATACGGGAGCTC 3′

Rab31-R 5′ TCGGTCGACTCAACAGCACCGGCGGCT 3′

Twist1,

Twist1-F 5′ CGGGAGTCCGCAGTCTTA 3′

Twist1-R 5′ GCTTGAGGGTCTGAATCTTG 3′

GAPDH,

GAPDH-F 5′ CCATGGAGAAGGCTGGGG 3′

GAPDF-R 5′ CAAAGTTGTCATGGATGACC 3′

### In vivo human PDX model based therapeutic study

Six-week-old BALB/c-nu male nude mice were obtained from the Shanghai Experiment Animal Center. PDX models were obtained as previously published reports [40, 41]. Randomization was conducted. PDX tumors with high expressed Rab31 were generated from STAD patients and immediately implanted. Tumor length (*L*, the longest diameter) and width (*W*, the shortest diameter) were measured weekly using a caliper and tumor volumes were calculated using the formula: (*L* × W^2^)/2. When the tumor reached an appropriate volume (50–100 mm^3^), the tumor-bearing mice were randomly divided into four groups (6 mice per group), and treated for 2 weeks. Control group, received PBS every 2 days via tail vein injection; siRNA group, Cholesterol-modified Rab31 siRNA (RiboBio, China) were intratumorally injected every 2 days. cisplatin, received cisplatin at 2.5 mg/kg every 2 days by tail vein injection; combination groups. Mice were sacrificed and tumors were dissected for staining (HE and IHC). All animal studies were approved by the Animal Care Ethics Committee of our institution and performed in accordance with the institutional guidelines.

### Hematoxylin–eosin (HE) staining

Tissues collected from the nude mice were fixed with 4% paraformaldehyde, embedded into paraffin, then stained with hematoxylin solution and eosin solution. After dehydration with graded ethanol, the slides were photographed using a Nanozoomer 2.0-RS fluorescence microscope (Hamamatsu, Japan).

### Statistical analysis

All data are presented as mean ± SD and were analyzed using GraphPad Prism (version 8; GraphPad, SanDiego, CA). Student’s t-test were used to analyze differences between groups, and *P* < 0.05 was considered statistically significant (*P < 0.05, **P < 0.01, and ***P < 0.001). Experiments were performed independently at least three times.

## Supplementary information

Supplementary Methods

Supplementary Figure 1

Supplementary Figure 2

Supplementary Figure 3

Supplementary Figure 4

Supplementary Figure 5

Supplementary Figure 6

reproducibility checklist

uncropped western blots Figure 1F

uncropped western blots Figure 2A

uncropped western blots Figure 2D

uncropped western blots Figure 2E F

uncropped western blots Figure 3A

uncropped western blots Figure 3C

uncropped western blots Figure 3D

uncropped western blots Figure 4A

uncropped western blots Figure 4D

uncropped western blots Figure 4E

uncropped western blots Figure 4F G H

uncropped western blots Figure 4K up

uncropped western blots Figure 4K down

uncropped western blots Figure S3

## Data Availability

All data needed to evaluate the conclusions in the paper are present in the paper. Additional data related to this paper are available from the corresponding author upon reasonable request.
