# Supplementary material for: Rab31 promotes metastasis and cisplatin resistance in stomach adenocarcinoma through Twist1-mediated EMT
Source: Cell Death Dis. 2023 Feb 13;14(2):115. doi: 10.1038/s41419-023-05596-4 (PMC9925739; doi:10.1038/s41419-023-05596-4)
Supplement: Supplementary file 1 — Supplementary Methods [file 41419_2023_5596_MOESM1_ESM.docx]

**Rab31 Promotes Metastasis and Cisplatin Resistance in Stomach Adenocarcinoma through Twist1-Mediated EMT**

**Supplementary methods**

**5-Ethynyl-2′-deoxyuridine (EdU) incorporation assay**

For the EdU proliferation assay, a Click-iT™ EdU Imaging kit (Invitrogen; Carlsbad, CA, USA) was used according to the manufacturer’s instruction. Briefly, cells were incubated with EdU in 96-well plates for 2 h. Thereafter, cells were fixed with 4% paraformaldehyde, permeabilized with 0.5% Triton X-100. Then, the cells were incubated with Click-iT reaction mixture, followed by Hoechst 33342. The results were observed using a NanoZoomer 2.0-RS fluorescence microscope (Hamamatsu, Japan).

**Co-immunoprecipitation assay**

The cells were harvested and lysed with IP buffer. Then lysates were centrifuged and incubated with protein A/G PLUS-Agarose beads (cat. no. sc-2003, Santa Cruz Biotech) at 4°C. Equal quantities of the lysates then incubated with normal mouse IgG (as a control) or specific antibody at 4 °C overnight, followed by incubated with protein A/G agarose for 1 h at room temperature. The beads were then washed five times and solubilized with loading buffer, followed by immunoblotting.

**Supplementary figure legends**

**Fig.S1** **Rab31 is associated with EMT. (A-F)** TWIST1, SNAI1, SNAI2, ZEB1 and ZEB2 were positively correlated with Rab31 in STAD.

**Fig.S2** **Rab31 regulates EMT by targeting MUC-1 in STAD. (A)** The interaction between Rab31 and Twist1/MUC-1 were tested using coimmunoprecipitation. (**B)** Immunohistochemical staining showed that Rab31 located both in cell cytoplasm and cell nuclear. (**C)** Twist1 was negatively correlated with MUC-1 in 375 samples. (**D)** Rab31 was negatively correlated with MUC-1 in 375 samples.

**Fig.S3** **Twist promotes migration and cisplatin resistance by targeting EMT in STAD. (A)** Transfection efficiency was assessed by Western Blotting in three STAD cell lines. (**B)** EMT markers in control group, cisplatin group and Twist siRNA plus cisplatin group were detected by western blot. (**C)** CCK-8 assays were performed to determine the sensitivity to cisplatin in the indicated groups. (**D, E**) Proliferation rates of STAD cells were analyzed using the EdU assay with an IC_50_ concentration of cisplatin. **p* < 0.05, ***p* < 0.01, ****p* < 0.001.

**Fig.S4 Rab31 promotes metastasis and depresses cisplatin sensitivity via EMT. (A)** STAD cells were transfected with control siRNA and Rab31 siRNA for 48 h, and the expression of indicated proteins were detected by western blot. **p* < 0.05, ***p* < 0.01 and ****p* < 0.001. (**B)** STAD cells were transfected with empty vector and Rab31 for 48 h, and the expression of indicated proteins were detected by western blot. **p* < 0.05, ***p* < 0.01 and ****p* < 0.001. (**C)** EMT markers in control group, cisplatin group and Rab31 siRNA plus cisplatin group were detected by western blot. **p* < 0.05 and ***p* < 0.01.

**Fig.S5** **Rab31 promotes Twist1-mediated EMT by targeting MUC-1. (A)** western blot showed that Rab31 and Twist1 were upregulated after cisplatin treatment in AGS cells. ***p* < 0.01 and ****p* < 0.001. (**B)** Western blot analysis of Twist1, Rab31 and MUC-1 for the indicated groups in AGS cells. ***p* < 0.01 and ****p* < 0.001. (**C)** Western blot analysis of MUC-1, Twist1 and Rab31 for the indicated groups in AGS cells. **p* < 0.05, ***p* < 0.01 and ****p* < 0.001. (**D)** Western blot analysis of p-Stat3, Stat3 and Rab31 for the indicated groups in AGS cells. **p* < 0.05, ***p* < 0.01 and ****p* < 0.001. (**E)** Western blot analysis of Stat3, Rab31 and MUC-1 for the indicated groups in AGS cells. ns: not significant. **p* < 0.05, ***p* < 0.01 and ****p* < 0.001. (**F)** Transfection efficiency was assessed by western blot. **p* < 0.05 and ***p* < 0.01.

**Fig.S6** **MiR15 and miR373 may play an important role in Rab31-Twist1 pathway. (A, B)** The mRNA expression of miRNAs, NF-κB, STAT3, AKT and ERK were detected by qRT-PCR in AGS cells. ***p* < 0.01 and ****p* < 0.001.

**Supplementary Table 1:** The correlation between Rab31 and EF-TFs

| **Spearman correlation**  **coefficient with Rab31** | **p−value** | **R** |
| --- | --- | --- |
| ZEB1 | 1.7e−177 | 0.85 |
| ZEB2 | 4.9e−126 | 0.78 |
| TWIST1 | 2e−120 | 0.77 |
| SNAI2 (Slug） | 1.9e−91 | 0.70 |
| SNAI1 (Snail) | 8.9e−80 | 0.66 |

Note：EMT-TFs, Epithelial-mesenchymal transition transcription factors.
