# Supplementary figures and images for: Rab31 promotes metastasis and cisplatin resistance in stomach adenocarcinoma through Twist1-mediated EMT
Source: Cell Death Dis. 2023 Feb 13;14(2):115. doi: 10.1038/s41419-023-05596-4 (PMC9925739; doi:10.1038/s41419-023-05596-4)

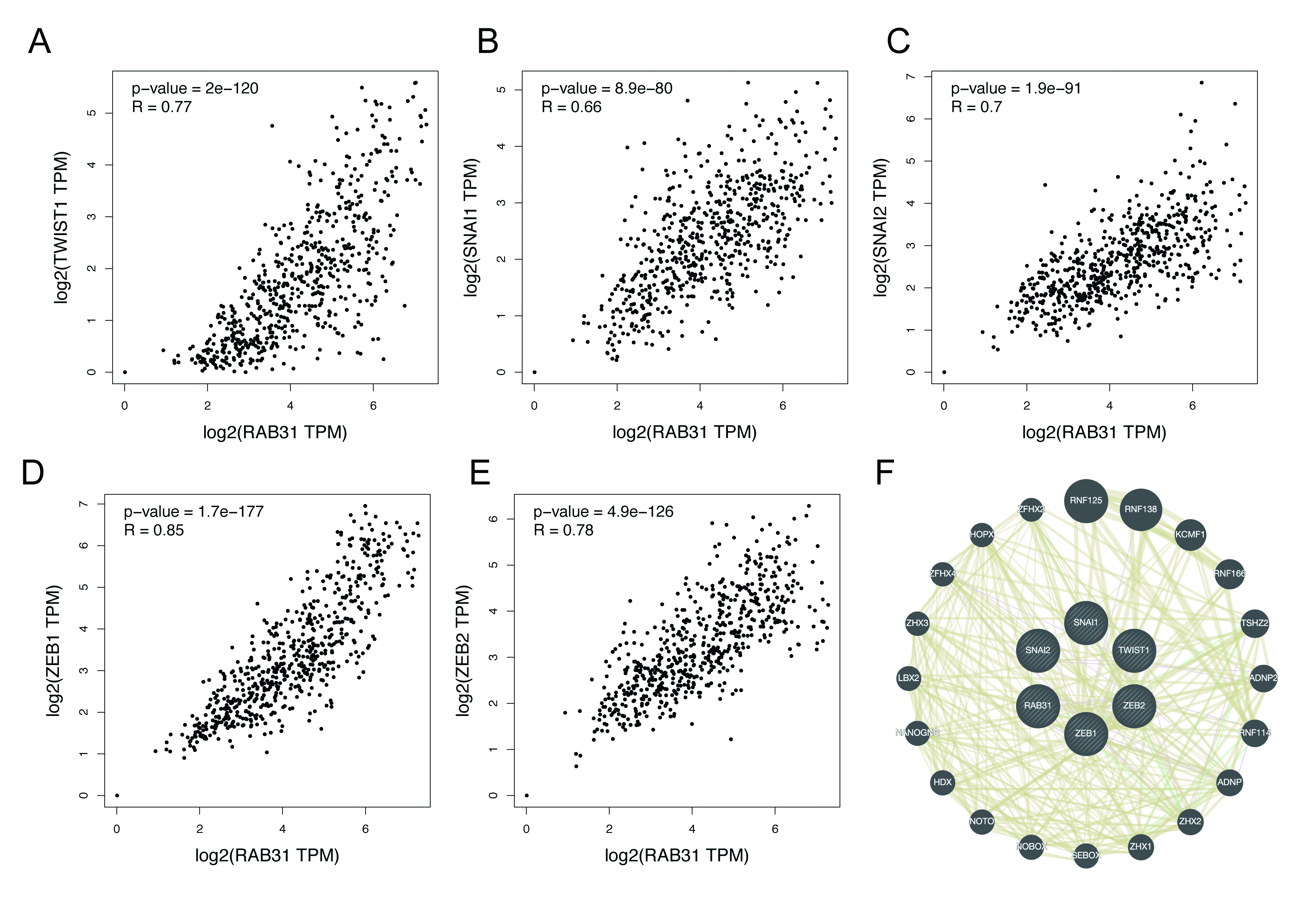

Supplement: Supplementary file 2 — Supplementary Figure 1 [file 41419_2023_5596_MOESM2_ESM.tif]

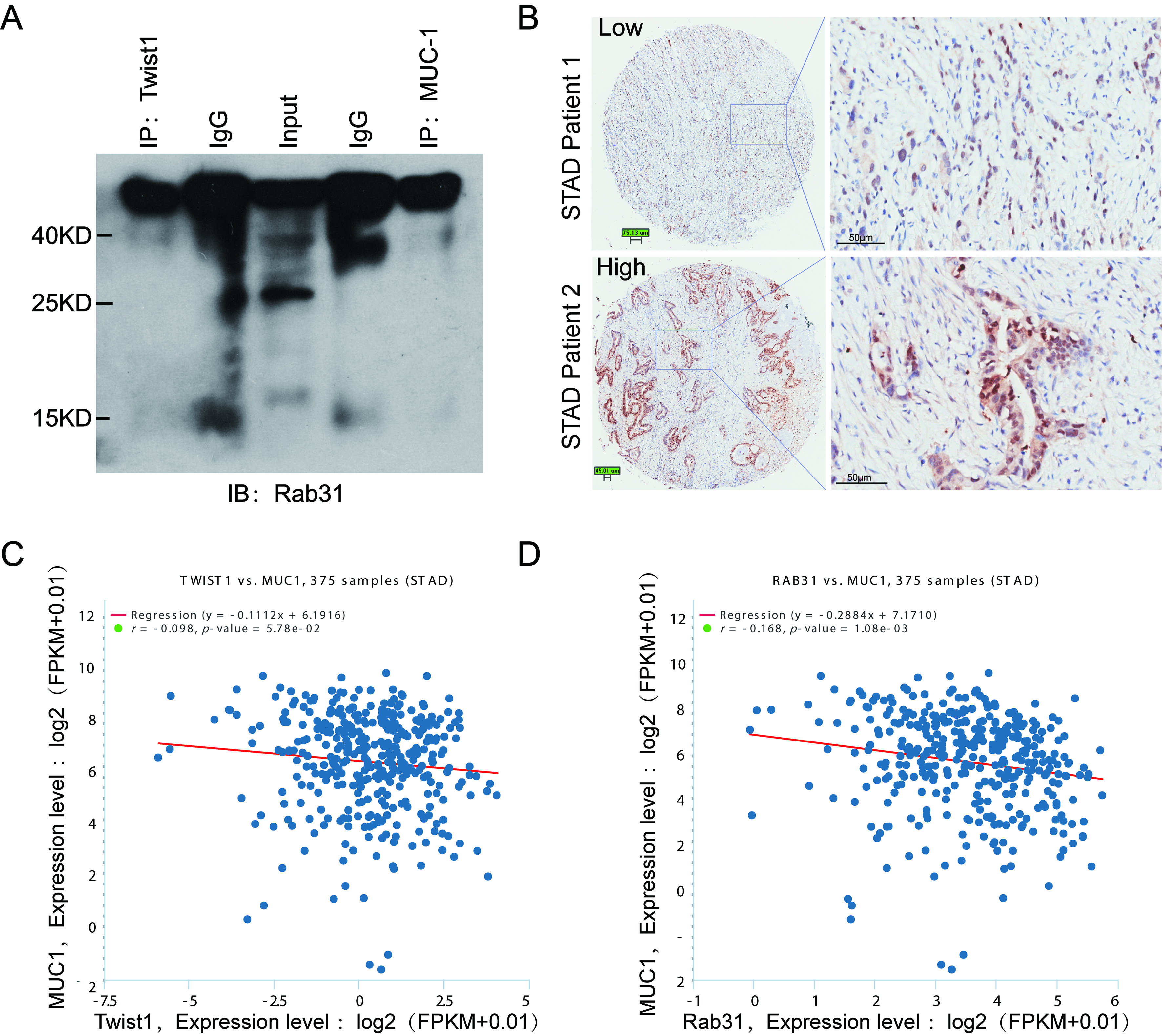

Supplement: Supplementary file 3 — Supplementary Figure 2 [file 41419_2023_5596_MOESM3_ESM.tif]

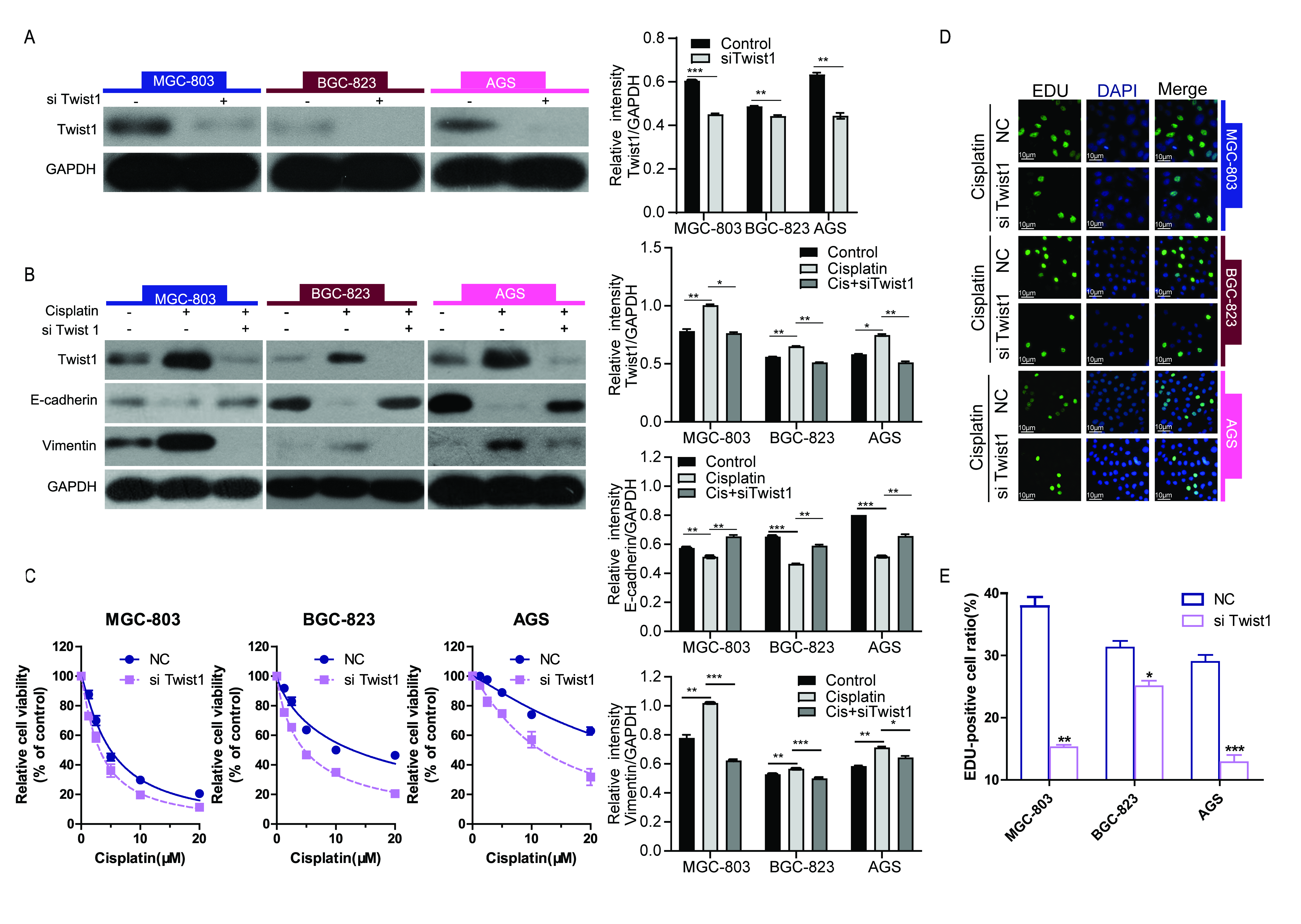

Supplement: Supplementary file 4 — Supplementary Figure 3 [file 41419_2023_5596_MOESM4_ESM.tif]

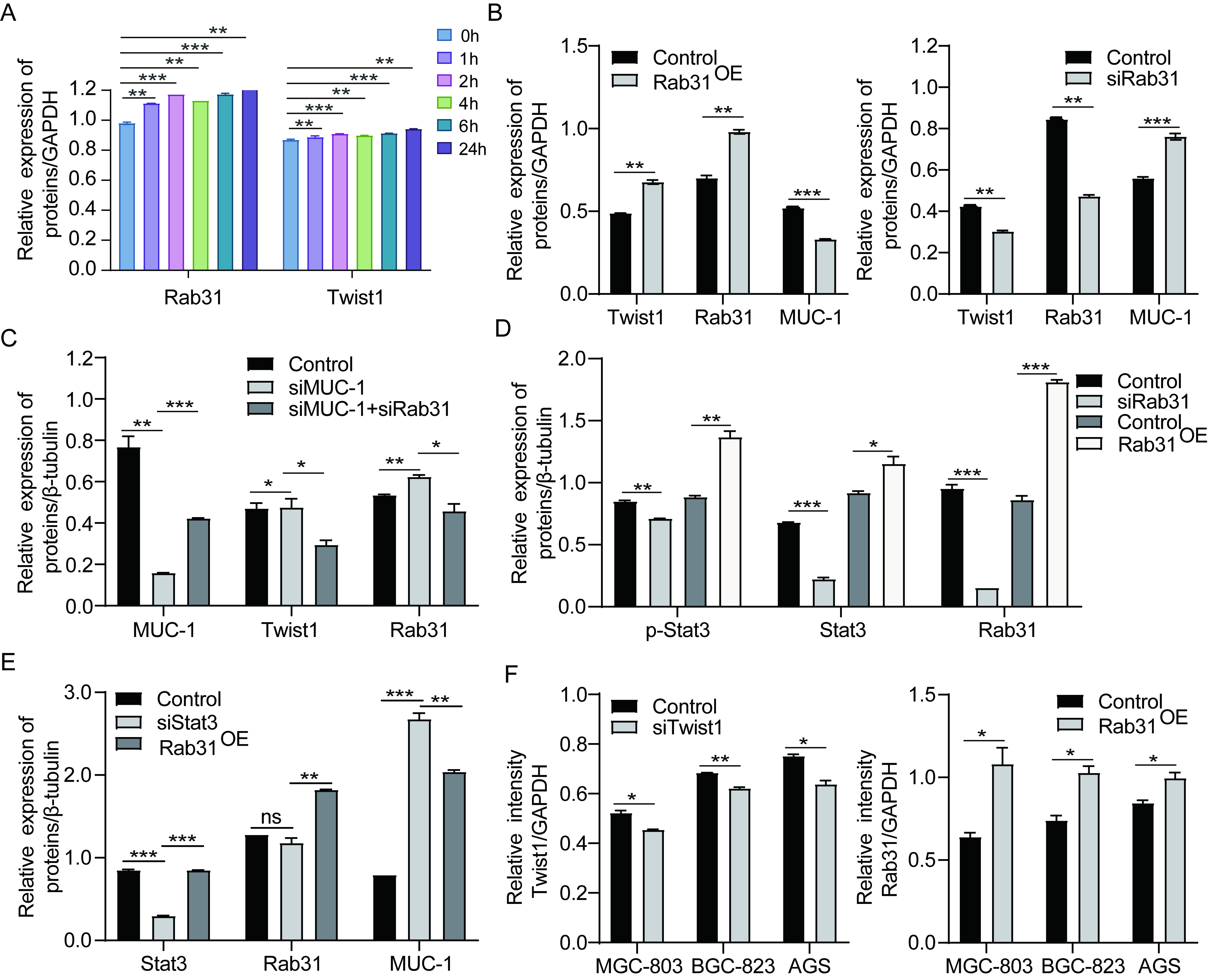

Supplement: Supplementary file 6 — Supplementary Figure 5 [file 41419_2023_5596_MOESM6_ESM.tif]

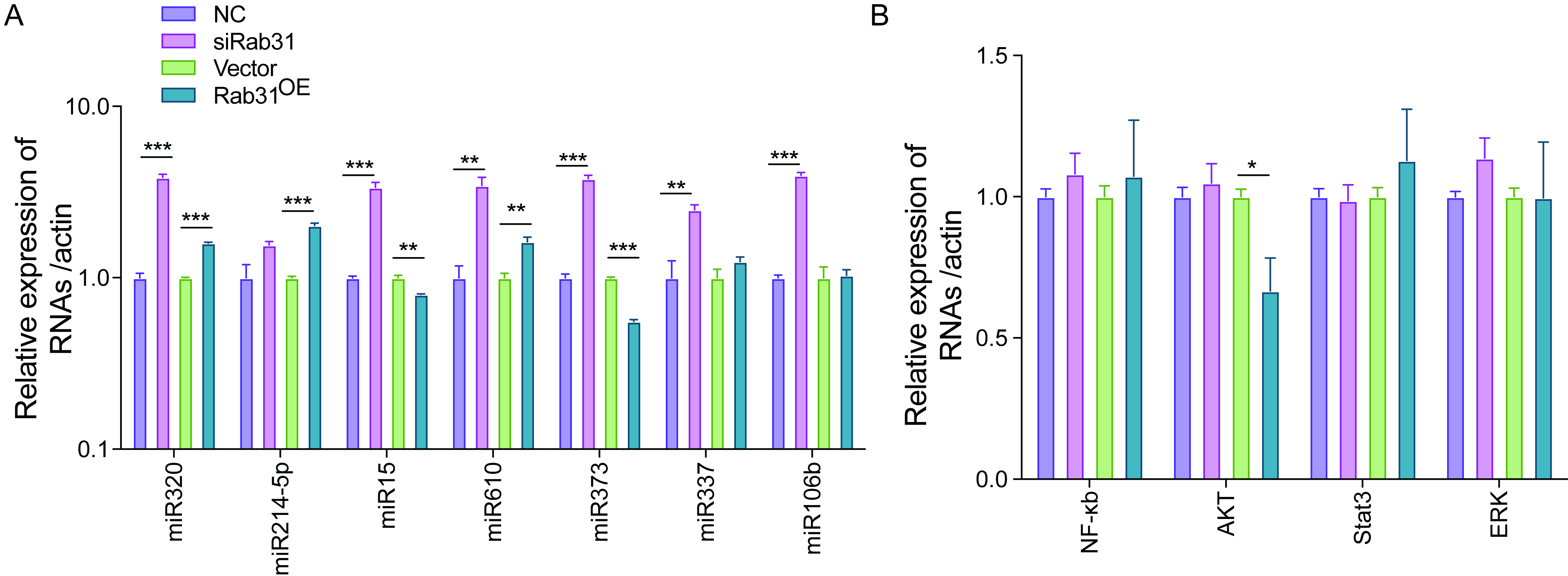

Supplement: Supplementary file 7 — Supplementary Figure 6 [file 41419_2023_5596_MOESM7_ESM.tif]

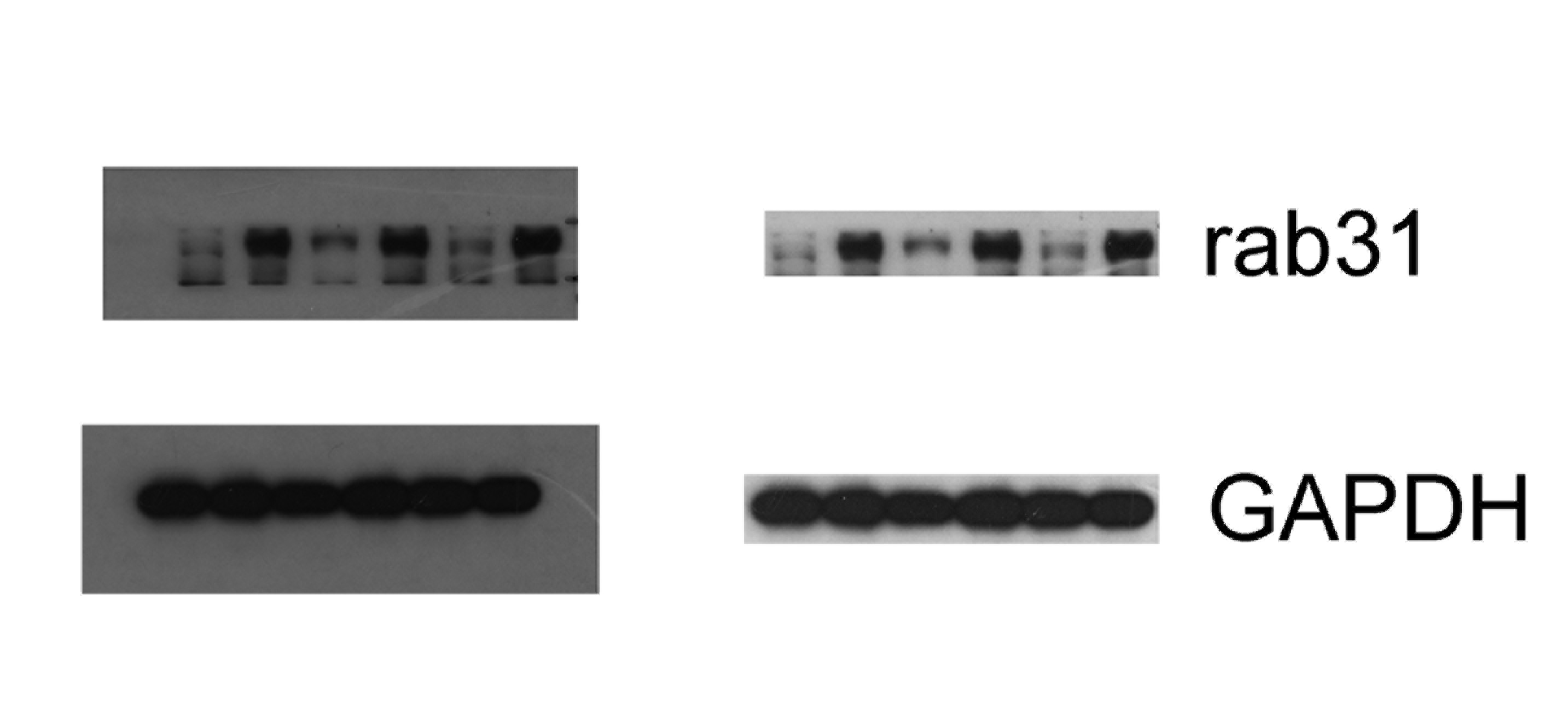

Supplement: Supplementary file 9 — uncropped western blots Figure 1F [file 41419_2023_5596_MOESM9_ESM.tif]

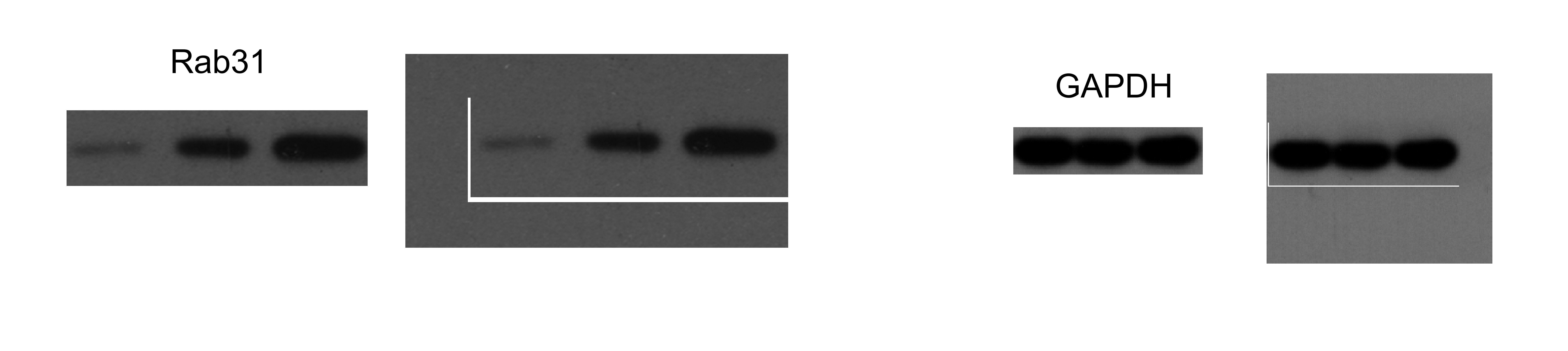

Supplement: Supplementary file 10 — uncropped western blots Figure 2A [file 41419_2023_5596_MOESM10_ESM.tif]

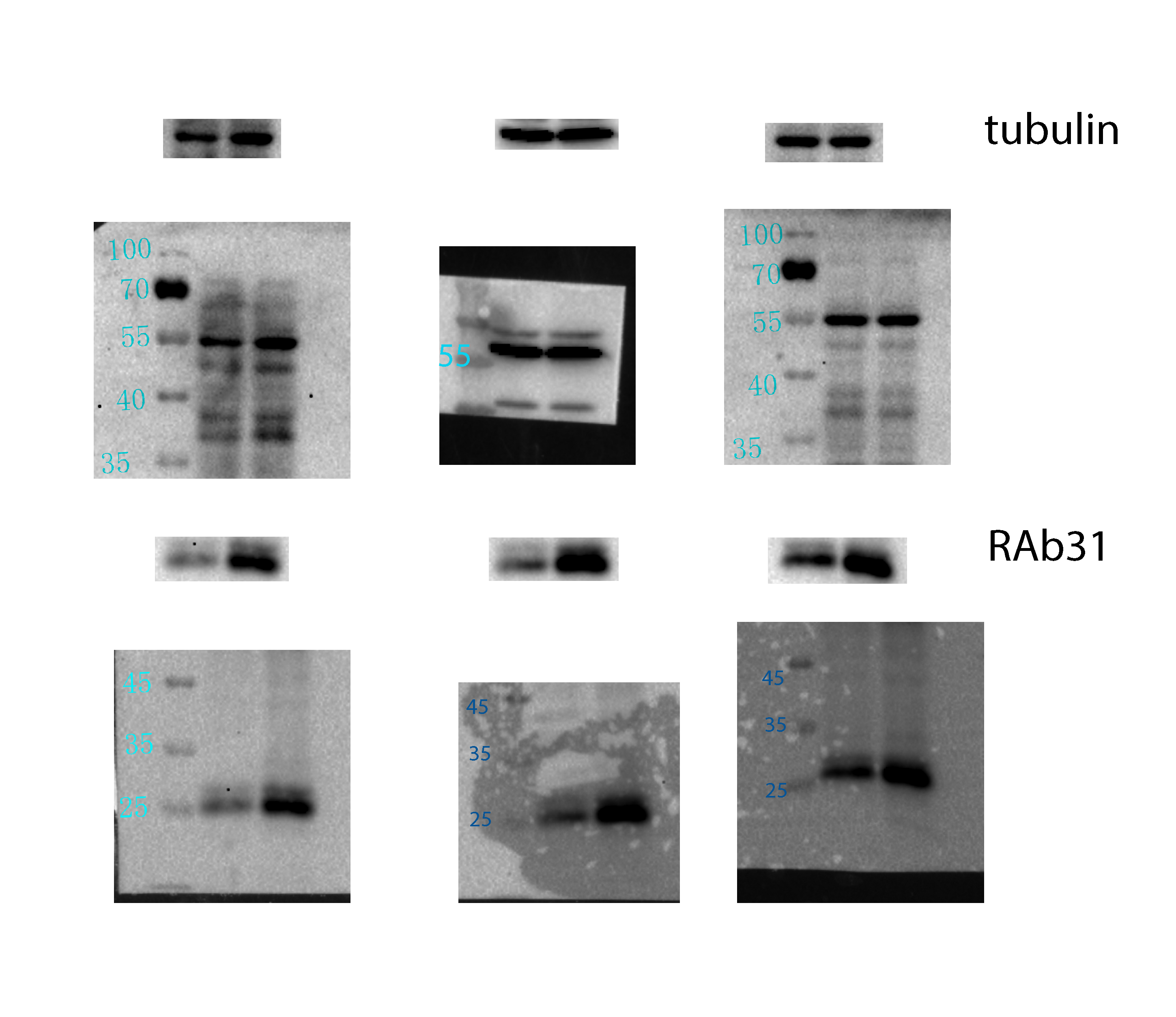

Supplement: Supplementary file 11 — uncropped western blots Figure 2D [file 41419_2023_5596_MOESM11_ESM.tif]

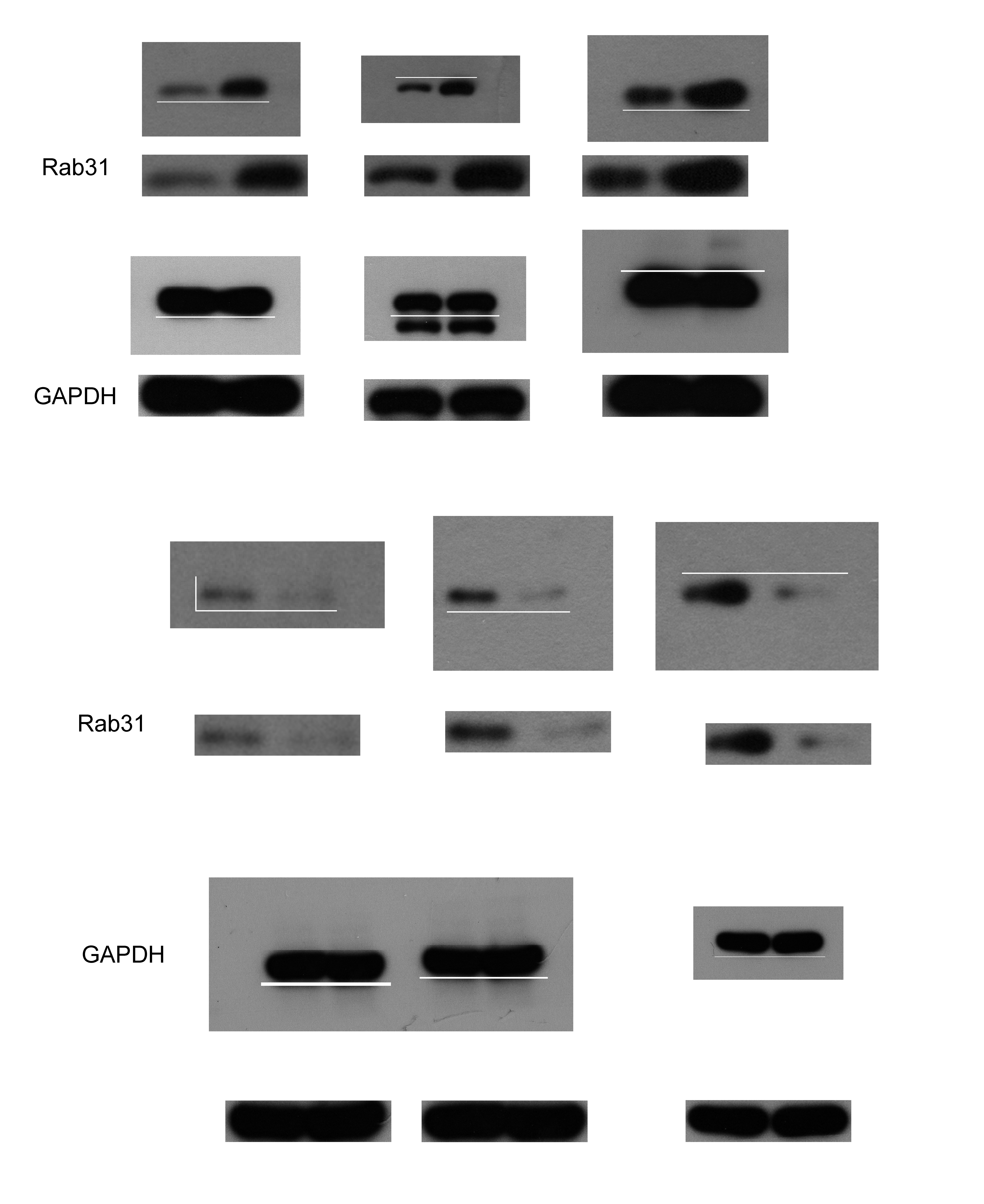

Supplement: Supplementary file 12 — uncropped western blots Figure 2E F [file 41419_2023_5596_MOESM12_ESM.tif]

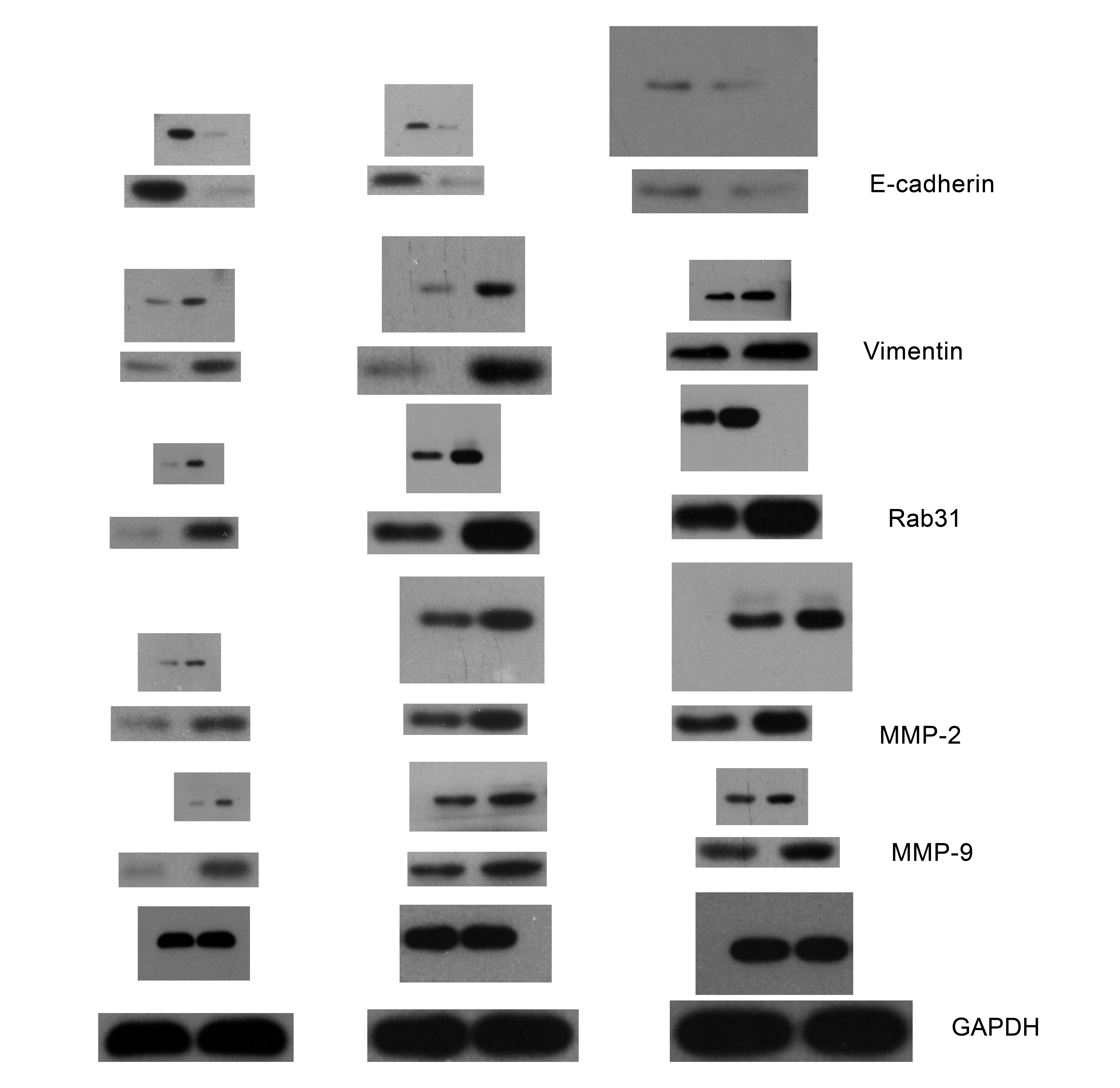

Supplement: Supplementary file 14 — uncropped western blots Figure 3C [file 41419_2023_5596_MOESM14_ESM.tif]

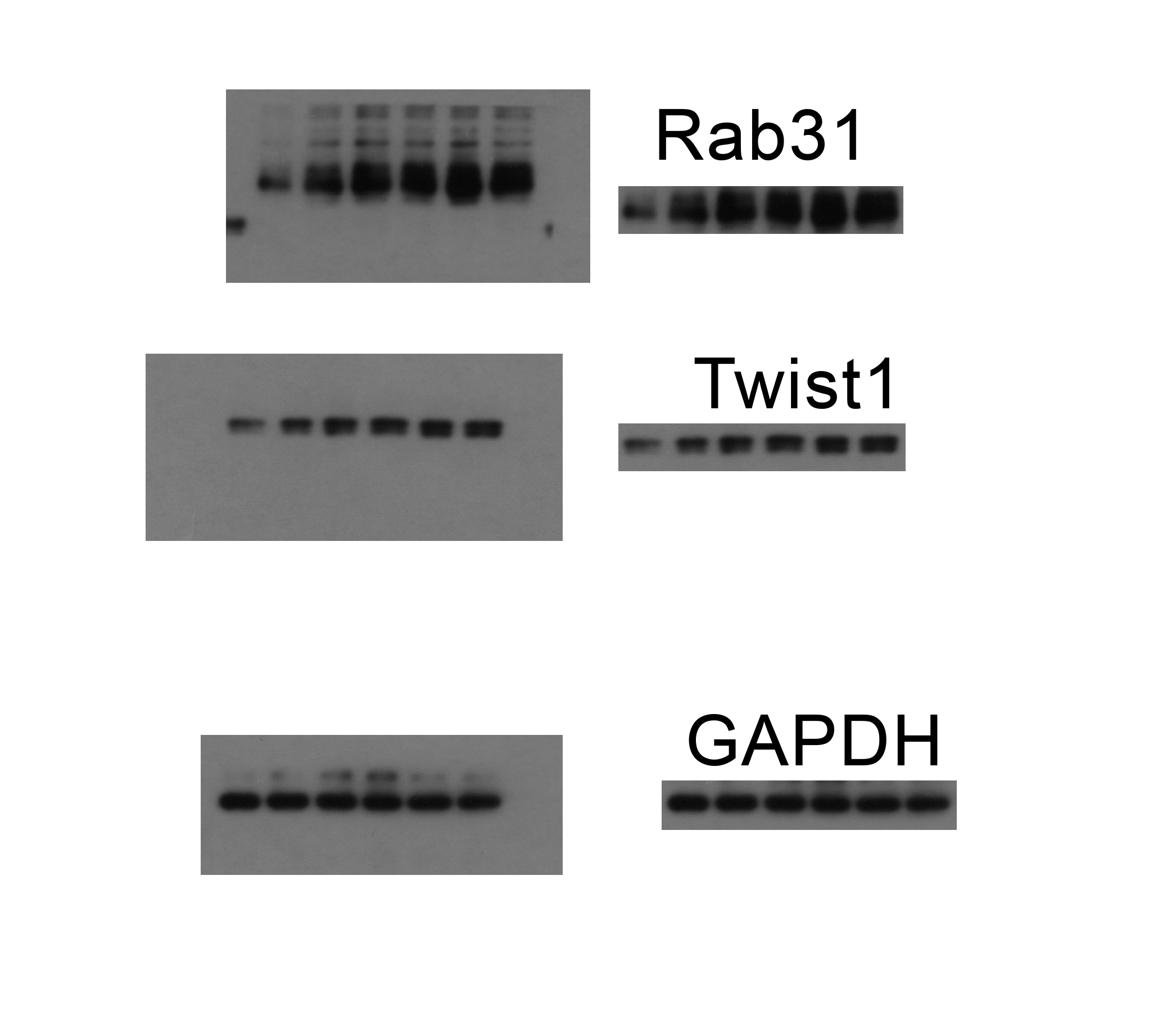

Supplement: Supplementary file 16 — uncropped western blots Figure 4A [file 41419_2023_5596_MOESM16_ESM.tif]

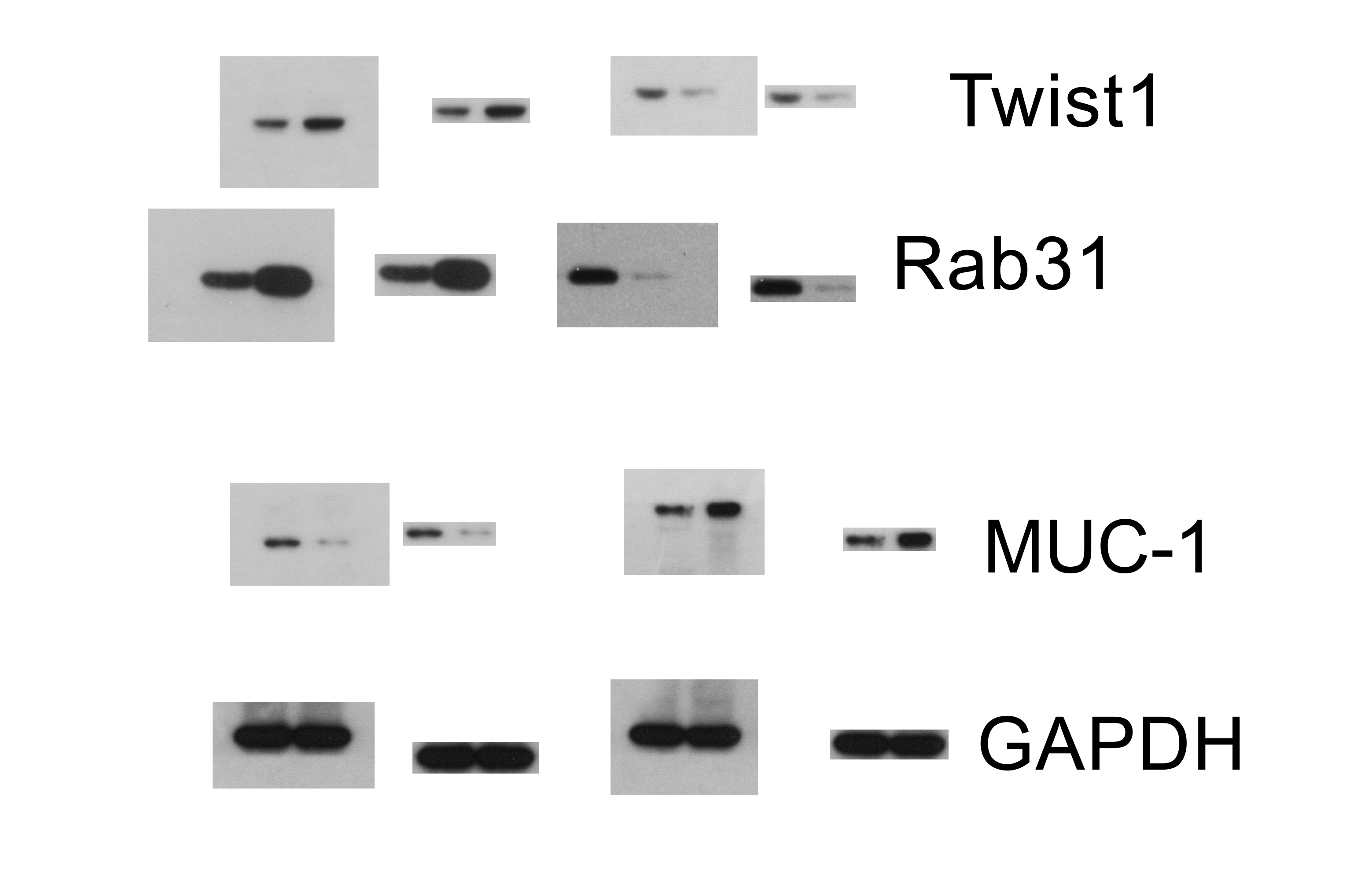

Supplement: Supplementary file 17 — uncropped western blots Figure 4D [file 41419_2023_5596_MOESM17_ESM.tif]

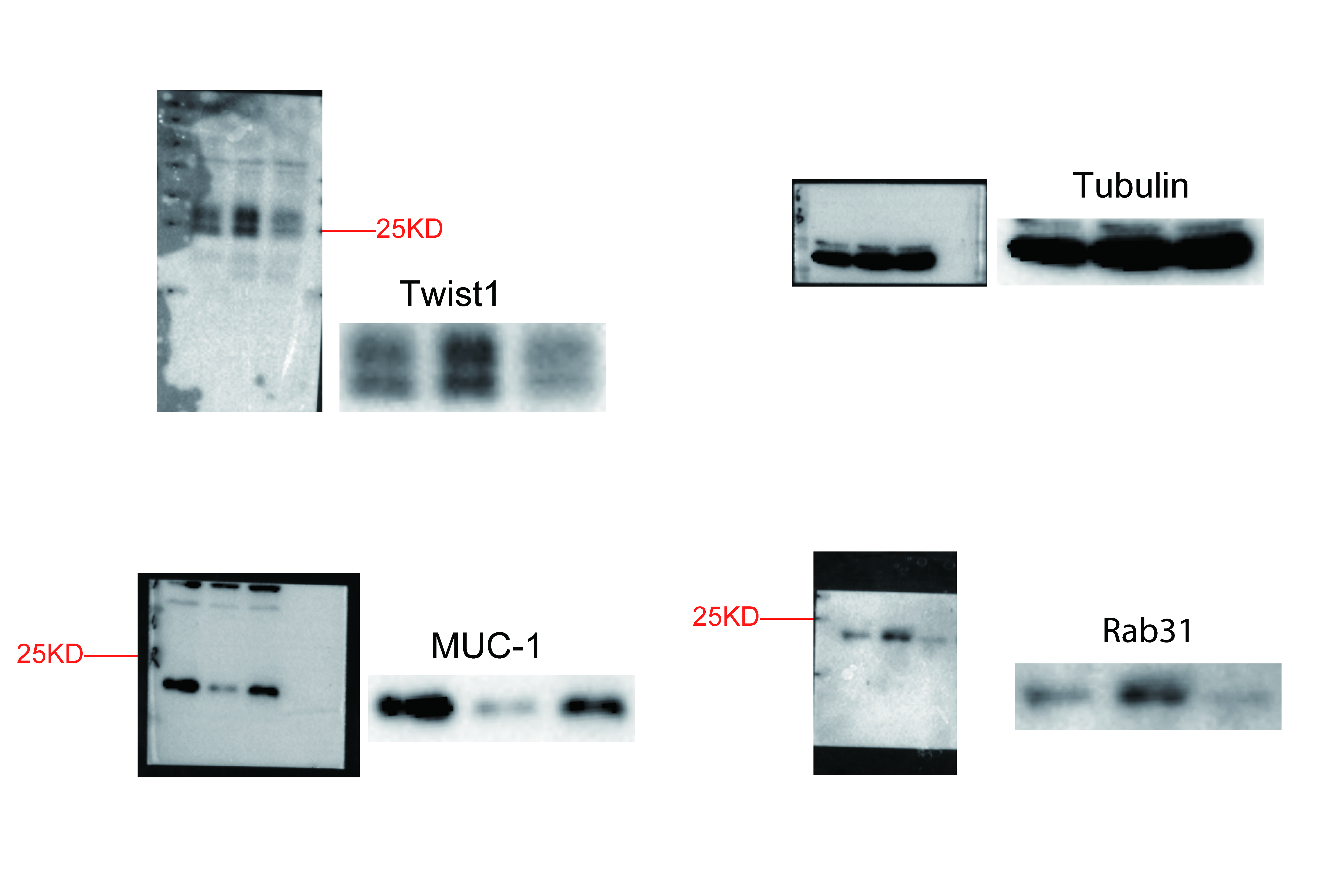

Supplement: Supplementary file 18 — uncropped western blots Figure 4E [file 41419_2023_5596_MOESM18_ESM.tif]

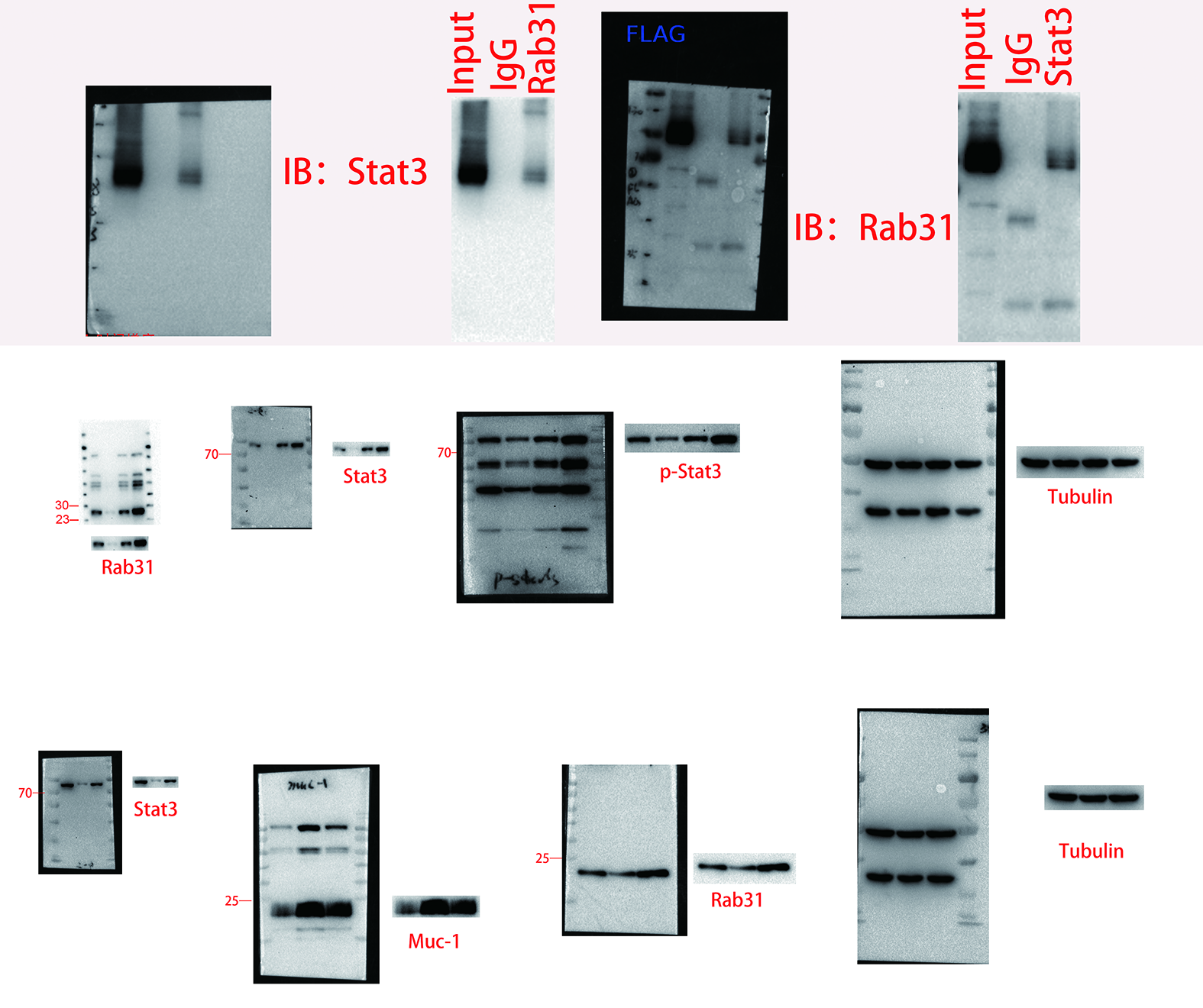

Supplement: Supplementary file 19 — uncropped western blots Figure 4F G H [file 41419_2023_5596_MOESM19_ESM.tif]

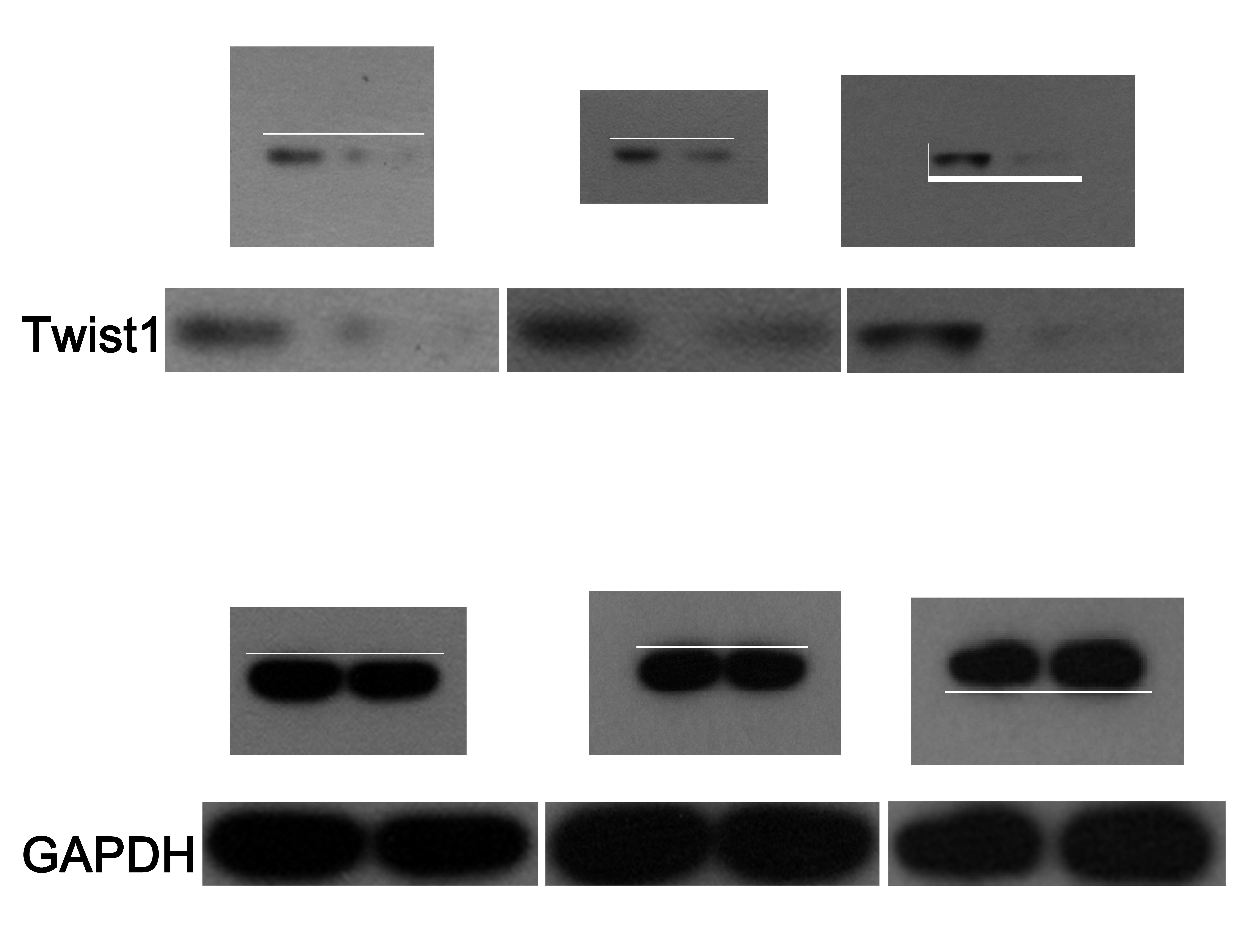

Supplement: Supplementary file 20 — uncropped western blots Figure 4K up [file 41419_2023_5596_MOESM20_ESM.tif]

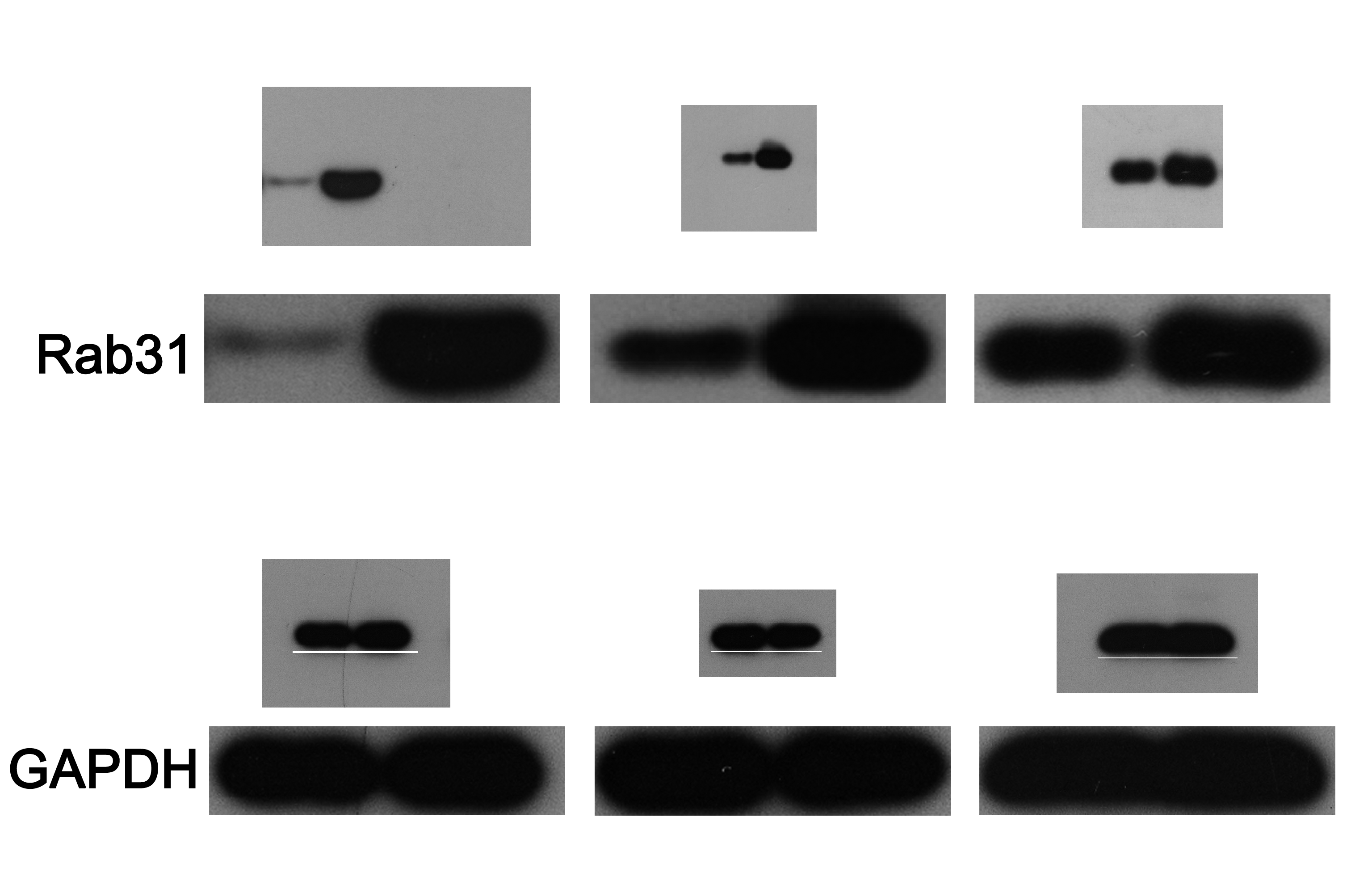

Supplement: Supplementary file 21 — uncropped western blots Figure 4K down [file 41419_2023_5596_MOESM21_ESM.tif]
